# To elucidate the effect of Ruanjian Qingmai granules on arteriosclerosis obliterans from the perspective of cholesterol efflux

**DOI:** 10.3389/fmed.2025.1510927

**Published:** 2025-08-04

**Authors:** Chenglin Jia, Biying Hong, Yujie Jiang, Chao Ma, Wei Liu, Yicheng Xu, Jian Chen, Yan Xie, Guangbo Ge, Ye-min Cao, Tianhua Yan, Yongbing Cao

**Affiliations:** ^1^Shanghai TCM-Integrated Hospital, Institute of Vascular Diseases, Shanghai University of Traditional Chinese Medicine, Shanghai, China; ^2^Department of Basic Medicine and Clinical Pharmacy, China Pharmaceutical University, Nanjing, China; ^3^Shuguang Hospital, Shanghai University of Traditional Chinese Medicine, Shanghai, China; ^4^Research Center for Health and Nutrition, Shanghai University of Traditional Chinese Medicine, Shanghai, China; ^5^Institute of Interdisciplinary Integrative Medicine Research, Shanghai University of Traditional Chinese Medicine, Shanghai, China

**Keywords:** arteriosclerosis obliterans, Ruanjian Qingmai granules, lipid reprogramming, *CYP7A1*, potential bioactive components

## Abstract

**Aim of the study:**

To investigate the effect of Ruanjian Qingmai granules (RJQM) on arteriosclerotic obliterans (ASO) and identify its potential bioactive components.

**Materials and methods:**

Separate zebrafish atherosclerosis models and cellular lipid metabolism disorder models were established, and RJQM was administered at different concentrations for intervention. The lipid deposition was examined by using Nile Red staining. The expression levels of cholesterol metabolism-related genes were determined by using quantitative real-time PCR (qRT-PCR). The *CYP7A1* inhibitor was utilized to elucidate the target of RJQM. Through network pharmacology and serum pharmacochemistry approaches, potential bioactive components were systematically identified and subsequently validated through experimental assays.

**Results:**

Ruanjian Qingmai granules significantly decreased lipid deposition and significantly increased the expression of *CYP7A1* mRNA in both zebrafish and HepaRG cells. And this effect was attenuated by *CYP7A1* inhibitors. Serum pharmacochemistry and network pharmacological analysis indicated that kaempferol and isorhamnetin were potential bioactive components in RJQM for the treatment of ASO. Both components could significantly reduce lipid deposition in zebrafish and HepaRG cells, and this effect was also diminished by *CYP7A1* inhibitors. Molecular docking also confirmed that *CYP7A1* might be the target of kaempferol and isorhamnetin, and qRT-PCR results also verified that both components could significantly up-regulate the mRNA expression level of *CYP7A1*.

**Conclusion:**

Ruanjian Qingmai granules exerts a therapeutic effect on ASO by up-regulating the expression level of *CYP7A1* mRNA, thereby reprogramming lipid metabolism. Kaempferol and isorhamnetin are likely the main active components of RJQM in lipid metabolic reprogramming.

## 1 Introduction

Arteriosclerotic obliterans (ASO) is a prevalent peripheral vascular disease characterized by atherosclerosis involving lower extremity arteries leading to arterial stenosis or occlusion and resulting in chronic ischemia changes. It ranks as the third most common atherosclerotic disease globally (1). The global number of ASO patients has reached as high as 200 million. Its incidence rises with age, and among people over 70 years old, the incidence of ASO can range from 15% to 20% (2, 3). For ASO patients with gangrene, the 5 years mortality rate is 70%, and the incidence of cardiovascular events is increased, with 824 deaths due to cardiovascular events occurring per 10,000 patients annually (4, 5). Consequently, the early treatment of ASO is of great clinical significance.

Hyperlipidemia, hypertension and diabetes are major risk factors for the development of ASO. Among these, the prevalence of dyslipidemia in China is as high as 41% (6), and reducing cholesterol levels has been shown to effectively delay ASO progression (7). Studies have indicated that approximately 60%–80% of ASO patients also have at least one form of coronary artery disease, and 50% of arterial diseases are closely associated with high cholesterol levels. In recent years, drug development targeting cholesterol metabolism has been evolving continuously (8, 9). Current therapeutic strategies include statins, which competitively inhibit cholesterol synthesis (10); ezetimibe, which blocks cholesterol absorption (11); ACAT inhibitors, which suppress triglyceride synthesis; niacin, which reduces LDL-C secretion; and PCSK9 inhibitors which enhance LDL-C uptake. Despite these advancements, the clinical efficacy of existing drugs remains suboptimal. Consequently, research into novel therapies for lipid metabolism reprogramming continues to be a major focus. By 2030, hyperlipidemia-related diseases are projected to account for nearly 23.6 million deaths globally (12), underscoring the urgent need for more effective and safer therapeutic options.

Numerous studies have pointed out that Traditional Chinese Medicine (TCM) plays an advantageous role in the treatment of chronic diseases. In TCM theory, the term “gangrene” is first documented in “Ling Shu,” which noted that it occurs on the toes and its appearance is red and black. Due to insufficient blood supply to the lower extremities, patients with ASO exhibit symptoms such as intermittent claudication, resting pain, cold limbs, cyanosis, and even necrosis, aligning with TCM classification of “gangrene.” Ruanjian Qingmai granules (RJQM) were developed by Xi Jiuyi, a late master of traditional Chinese and western medicine, based on his over 50 years of clinical experience. RJQM granules are composed of five types of herbs: 30 g of *Sedum sarmentosum Bunge* (Chuipencao, CPC), 15 g of *Sargassum pallidum (Turn.) C.Ag*. (Haizao, HZ), 10 g of *Siegesbeckia pubescens Makino* (Xiqiancao, XQC), 15 g of *Typha angustifolia L.* (Shengpuhuang, PH), and 30 g of *Ostrea rivularis Gould* (Duanmuli, DML).

Through extensive preliminary clinical observation studies, our research group has confirmed that RJQM can significantly improve the clinical symptoms of ASO patients (13), protect endothelial cells (14), and establish collateral circulation (15). However, research on the mechanisms underlying RJQM’s effects on cholesterol metabolism remains poorly understood, warranting further investigation.

Due to the complex multi-component interactions of TCM compounds and the incomplete understanding of their bioactive substances and mechanisms, modern research on TCM faces significant challenges. To address this issue, the integration of network pharmacology and serum pharmacochemistry has emerged as a transformative strategy. Network pharmacology adopts a holistic approach by constructing compound-target-disease networks to predict potential targets and pathways (16, 17), while serum pharmacochemistry focuses on identifying bioactive components through in vivo absorption analysis, thereby bridging the gap between traditional formulations and modern pharmacological validation (18). Therefore, this study will combine network pharmacology, molecular docking, and serum pharmacochemistry to screen the therapeutic targets of RJQM for ASO and its potential bioactive components.

Zebrafish are characterized by a short developmental cycle, transparent embryo, and ease of assessment. Moreover, the homology between zebrafish and human genes reaches as high as 70%. Consequently, they are frequently employed as a crucial animal model for screening new drugs and identifying novel therapeutic targets for various diseases (19). Given the high similarity between zebrafish and humans in the formation mechanism of dyslipidemia and hypercholesterolemia (20), this study utilized a zebrafish atherosclerosis model induced by a high-cholesterol diet (21). The aim was to further explore the anti-atherosclerosis effect of RJQM and its potential bioactive components through reducing lipid deposition. This research is expected to facilitate the broader clinical application of RJQM and provide new ideas for the development of lipid-lowering drugs.

## 2 Materials and methods

### 2.1 Preparation of RJQM extract powder

The herbs composing the RJQM crude drug were purchased from Shanghai Wanshicheng Pharmaceutical Co. Ten dosages of RJQM were taken, with a total drug amount of 1,000 g, namely 300 g of CPC, 150 g of HZ, 100 g of XQC, 150 g of PH, and 300 g of DML. First, add 10 times the amount of water to DML and extract it for 1 h. Then, the pre-boiled DML and its aqueous extract were combined with the other four herbs, which had been soaked for 45 min. Add 10 times the amount of water and extract for three times, each time for 1 h. Filter the mixture, and combine the filtrates to obtain the medicinal materials extract. Concentrate the extract by thin-film evaporation to a density of 1.04 g/mL, let it stand at 4°C for 24 h, take the supernatant and concentrate to 1.35 g/mL. Add dextrin in a 1:1 ratio, stir evenly, dry it under vacuum at 65°C, crush it, and sieve it through an 80-mesh sieve to obtain the RJQM extract powder, which was used in all animal experiments in this study.

### 2.2 Preparation of high-fat feed for zebrafish

According to an 8% mass ratio, zebrafish feed and cholesterol powder were precisely weighed and mixed. Ether was added to the mixture, which was placed in a water bath at 30°C. The mixture was fully dissolved and allowed to evaporate, resulting in the formation of high-fat feed. The feed was subsequently air-dried in a cool, ventilated environment and stored at 4°C.

### 2.3 Animal feeding

Male Wistar rats (200 g) were purchased from Sipur-Bikai Laboratory Animal Co., Ltd., Shanghai, China. They were housed in the Animal Laboratory Center of Shanghai University of Traditional Chinese Medicine under SPF conditions. The environmental parameters were maintained as follows: temperature at 25 ± 3°C, humidity at 55 ± 10%, and a 12 h light/dark cycle. The animals had ad libitum access to purified feed and water. Animal ethics approval number: PZSHUTCM2306270002.

Wild-type AB zebrafish eggs and Fli1a-EGFP transgenic zebrafish eggs were purchased from Yishulihua, Nanjing, China. They were housed in the Zebrafish Experimental Center, Institute of Vascular Disease, Shanghai Hospital of Integrated Traditional Chinese and Western Medicine. The rearing temperature was maintained at around 28 ± 0.5°C, with a light/dark cycle of 14:10 h. The zebrafish larvae were fed twice a day.

### 2.4 Cell culture

Human hepatoma cell line, HepaRG, was donated by Professor Li Ling’s research group of Shanghai TCM-Integrated Vascular Anomalies Institute. HepaRG cells were cultured in Dulbecco’s Modified Eagle Medium (DMEM), supplemented with 10% fetal bovine serum (FBS) and 1.5% penicillin-streptomycin. The cells were maintained in a humidified incubator at 37°C with 5% CO_2_ to ensure optimal growth conditions.

### 2.5 Serum pharmacochemical analysis of RJQM

According to the method described in “2.1,” 71 g of extract powder can be obtained from 1,000 g of RJQM crude drug. Based on the body surface area conversion method, Wister rats were administered a single dose of RJQM equivalent to twice the clinical dose (1.28 g/Kg).

Considering that different components in TCM formulas exhibit distinct absorption peaks, blood samples were collected from Wister rats before and at 0.5, 1, and 2 h after intragastric administration to avoid missing important active components. The blood samples were centrifuged, and the supernatant was collected. Five times the volume of methanol was added to precipitate the protein, and centrifuged at 10,000 × *g* for 15 min (4°C). The supernatants were dried under nitrogen at 37°C, redissolved with 20% methanol solution, centrifuged at 10,000 × *g* for 15 min, and the supernatant was taken for analysis. RJQM extract was diluted with 20% methanol and then filtered through 0.22 μm membrane to obtain a test solution of the RJQM extract with a concentration of 35 mg/mL.

The components of the RJQM extract solution and supernatant samples were analyzed using a Dionex Ultimate 3,000 high-performance liquid chromatography system (Thermo Fisher Scientific, Massachusetts, United States) and was equipped with an Acquity UPLC BEH C18 column (100 × 2.1 mm, 1.7 μm; Waters, Massachusetts, United States).

The mobile phase consisted of 0.1% formic acid in water and methanol, with gradient elution at a flow rate of 0.3 mL/min. The column temperature was maintained at 40°C, and the injection volume was 2 μL. Analysis was performed using a Q Exactive hybrid quadrupole-Orbitrap mass spectrometer equipped with an electrospray ionization source. The scanning mode utilized was the positive and negative ion full MS/SIM mode. The mass resolution was set to 70,000 full width at half maximum (FWHM), with a mass scan range of m/z 80–1,200 for both ionization modes.

### 2.6 Preparation of RJQM drug-containing serum

Ten Wistar rats were randomly divided into a control group and an RJQM group. The RJQM group was administered RJQM extract at a dose of 1.28 g/Kg by gavage twice a daily (morning and evening) for five consecutive days, while the control group received an equivalent volume of PBS. One hour after the final administration (following a 12 h fasting period with free access to water), blood samples were collected from the abdominal aorta under gas anesthesia. The collected blood was allowed to clot at 37°C for 1 h and then centrifuged at 3,000 r/min for 10 min. The supernatant serum was collected, heat-inactivated in a water bath at 56°C for 30 min, and subsequently filtered through a 0.22 μm membrane. Thus, the RJQM-containing serum and control serum were obtained and stored at −20°C for subsequent analysis.

### 2.7 Construction of the atherosclerosis model and drug intervention in zebrafish

Wild-type zebrafish with healthy development of up to 5 days post fertilization (dpf) were fed a high-fat diet containing 8% cholesterol twice daily (Fli1a-EGFP transgenic zebrafish were fed with 8% fluorescent cholesterol-containing diet), to establish the zebrafish atherosclerosis model (22). The RJQM low, medium, and high groups (abbreviated as RJQML, RJQMM, RJQMH, respectively) were co-treated with different concentrations of RJQM (0.025, 0.05 and 0.1 mg/ml/d) for 5 or 15 days. The control group was given a normal diet.

Furthermore, a *CYP7A1* inhibitor group (using obeticholic acid, abbreviated as OCA, with a final concentration of 4 μM), kaempferol low, medium, and high-dose groups (abbreviated as KPFL, KPFM, KPFH, respectively), and isorhamnetin low, medium, and high-dose groups (abbreviated as ISOL, ISOM, ISOH, respectively) were set up. They were given 1, 5 and 10 μg/ml/d of kaempferol or isorhamnetin, respectively.

### 2.8 Cell experiment

The lipid deposition model of HepaRG cells was established by treating the cells with 0.25 mM oleic acid (OA) (23). The control was treated with 10% control serum and the model groups were treated with 10% control serum plus 0.25 mM OA. The RJQML, RJQMM, and RJQMH groups were co-treated with 0.25 mM OA + 5%, 10%, or 20% RJQM-containing serum for 24 h. Additionally, the experimental design included: (1) an OCA group, co-treated with 0.25 mM OA + 30 μM OCA); (2) an OCA + RJQMM group, co-treated with 0.25 mM OA + 30 μM OCA + 10% RJQM-containing serum; (3) kaempferol treatment groups (KPFL, KPFM, KPFH), co-treated with 0.25 mM OA + 5, 10, and 20 μM kaempferol, respectively; (4) isorhamnetin treatment groups (ISOL, ISOM, ISOH), co-treated with 0.25 mM OA + 5, 10, and 20 μM isorhamnetin, respectively; and (5) combination treatment groups (KFMM + OCA and ISOM + OCA), co-treated with 0.25 mM OA + 30 μM OCA + 10 μM kaempferol or isorhamnetin.

### 2.9 Evaluation of Nile Red staining and lipid deposition

A total of 1.3 mg/mL Nile Red dye solution (McLean, Shanghai, China) was used to incubate zebrafish or the fixed cells for 30 min in the dark, followed by PBS cleaning. Zebrafish were anesthetized using 0.03% tricaine methanesulfonate and immobilized in 3% (w/v) sodium carboxymethyl cellulose. Lipid deposition in zebrafish was observed using a nanoscale ultra-high laser confocal microscopy system (Leica STELLARIS STED, Wetzlar, Germany) with excitation wavelengths of 528 nm or dual excitation at 488 nm and 528 nm, respectively, using a 10 × objective for full scan.

For cellular analysis, after PBS washing, cells were stained with DAPI solution (C1005, Beyotime Biotechnology, Shanghai, China) for 10 min in the dark. Fluorescence imaging and quantitative analysis were performed using an ImageXpress Micro Confocal High-Content Screening System (Molecular Devices, Shanghai, China) equipped with a 20 × objective lens.

### 2.10 Quantitative reverse transcription-PCR (qRT-PCR)

Total RNA was extracted from zebrafish or cell samples using 1 ml TRIzol reagent followed by purification with a SteadyPure Universal RNA Extraction Kit (Accurate Biology, Shanghai, China). The concentration and purity were determined. cDNA synthesis was performed using Evo M-MLV Reverse Transcription premix Kit (Accurate Biology, Shanghai, China) with the following thermal conditions: 37°C for 15 min followed by 85°C for 5 s. The expression of mRNA related to cholesterol metabolism was detected using a SYBR Green Pro Taq Hs Premixed qPCR Kit (Accurate Biology, Shanghai, China). The reaction conditions of qPCR amplification were as follows: initial denaturation at 95°C for 30 s, followed by 40 cycles of denaturation at 95°C for 5 s and annealing /extension at 60°C for 15 s. The primer sequences used in this study are provided in Supplementary Material 1.

### 2.11 Network pharmacological analysis

The active components of RJQM were identified through comprehensive screening using established pharmacology databases and TCM analysis platforms, including the TCM systems Pharmacology Database (TCMSP^1^), HERB^2^, PubChem (see text footnote 1), and SwissTargetPrediction^3^. Disease-related targets were obtained by searching the keywords “arteriosclerosis obliterans,” “atherosclerosis,” and “hypercholesterolemia” in GeneCards (version 5.9) and DisGeNET (version 6.0), which represent comprehensive repositories of human disease-related genes and genetic variations, respectively. The intersection of drug and disease targets was visualized using Venny 2.1^4^, a bioinformatics tool for comparative analysis. Furthermore, the Kyoto Encyclopedia of Genes and Genomes (KEGG) pathway enrichment analysis was performed using the DAVID database (version 6.8^5^).

### 2.12 Composition-target molecular docking

The three-dimensional (3D) crystal structure of target protein was retrieved from the Protein Data Bank (PDB)^6^. The molecular structures of active components identified through RJQM network pharmacology analysis were obtained from Pubchem database^7^, which provided comprehensive chemical information for blood component analysis of RJQM. Molecular docking analysis was performed using CB-Dock2^8^, an online platform protein-ligand interaction prediction.

### 2.13 Statistical analysis

SPSS 19.0 software was used for statistical analysis, and GraphPad Prism 8 software was used for graphing. The quantitative data are represented by mean ± standard deviation. The qRT-PCR data were processed using the 2^–ΔΔ^*^CT^* method. A one-way analysis of variance (ANOVA) was used to assess intergroup differences. The level of statistical significance was set at *p* < 0.05.

## 3 Results

### 3.1 RJQM reduced lipid deposition in zebrafish

**FIGURE 1:**
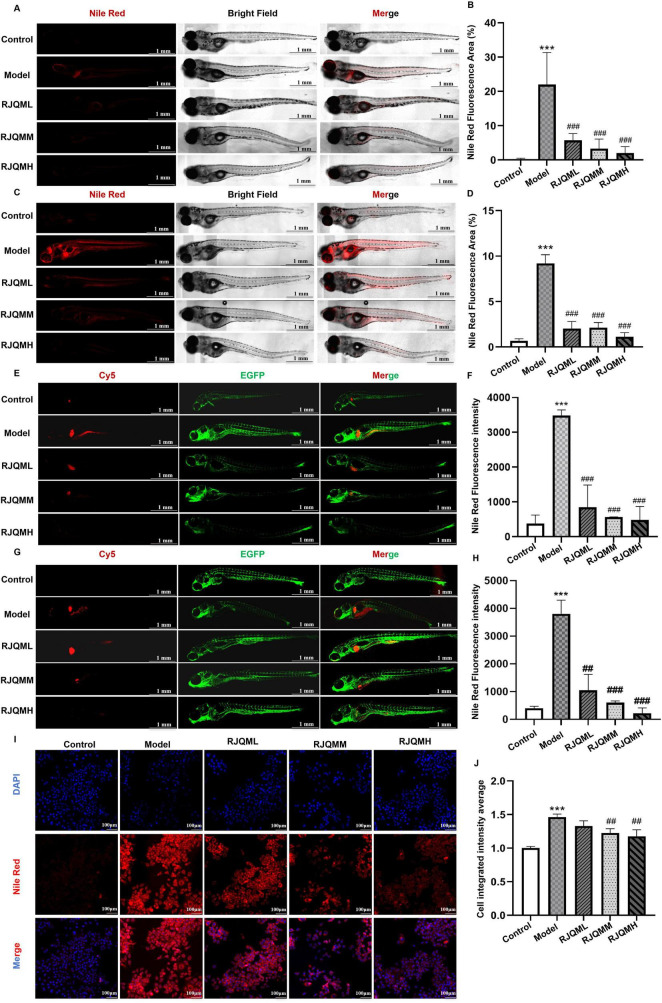
Ruanjian Qingmai granules (RJQM) significantly reduces lipid deposition in zebrafish and cellular models. **(A,B)** Representative Nile Red staining images **(A)** and fluorescence quantification **(B)** in wild-type AB zebrafish after 5 days treatments. **(C,D)** Representative Nile Red staining images **(C)** and fluorescence quantification **(D)** in wild-type AB zebrafish after 15 days treatments (*n* = 5). **(E,F)** Nile Red staining **(E)** and fluorescence quantification **(F)** in *Fli1a*-EGFP transgenic zebrafish after 5 days treatment. **(G,H)** Nile Red staining **(G)** and fluorescence quantization **(H)** in *Fli1a*-EGFP transgenic zebrafish after 15 days treatment (*n* = 3). Control, normal diet; Model, 8% high-cholesterol diet; RJQML, RJQMM, and RJQMH, co-treated with 8% high cholesterol diet + 0.025, 0.05, or 0.1 mg/ml/d RJQM. **(I)** Representative Nile Red staining images in HepaRG cells after 24 h treatment. **(J)** Quantitative analysis of Nile Red fluorescence intensity in HepaRG cells. Control, 10% control serum; Model, 10% control serum + 0.25 mM oleic acid (OA); RJQML, RJQMM, RJQMH, co-treated with 0.25 mM OA + 5%, 10%, or 20% RJQM drug-containing serum, respectively. ***p < 0.001 compared with the control group; ^##^*p* < 0.01, ^###^*p* < 0.001 compared with the model group.

As shown in Figures 1A, B, zebrafish fed an 8% cholesterol diet for 5 days exhibited significant lipid accumulation (*p* < 0.05). Treatment with RJQML, RJQMM and RJQMH significantly reduced lipid deposition in zebrafish after 5 days of intervention (*p* < 0.05). Persistent lipid deposition was observed following 15 days of high-cholesterol diet feeding, and all RJQM doses demonstrated significant lipid-reducing effects (Figures 1C, D). These findings were further confirmed using *Fli1a-EGFP* transgenic zebrafish (Figures 1E–H).

Considering the liver’s central role in lipid metabolism, we established a lipid deposition model in HepaRG cells using 0.25 mM oleic acid (OA). Consistent with the *in vivo* results, all RJQM doses significantly reduced lipid deposition after 24 h of treatment (Figures 1I, J). Based on these findings, we hypothesize that RJQM may exert its anti-ASO effects through lipid metabolism reprogramming. The medium dose of RJQM (RJQMM) was selected for further verification.

### 3.2 RJQM reduced lipid deposition by up-regulating *CYP7A1* mRNA expression

**FIGURE 2:**
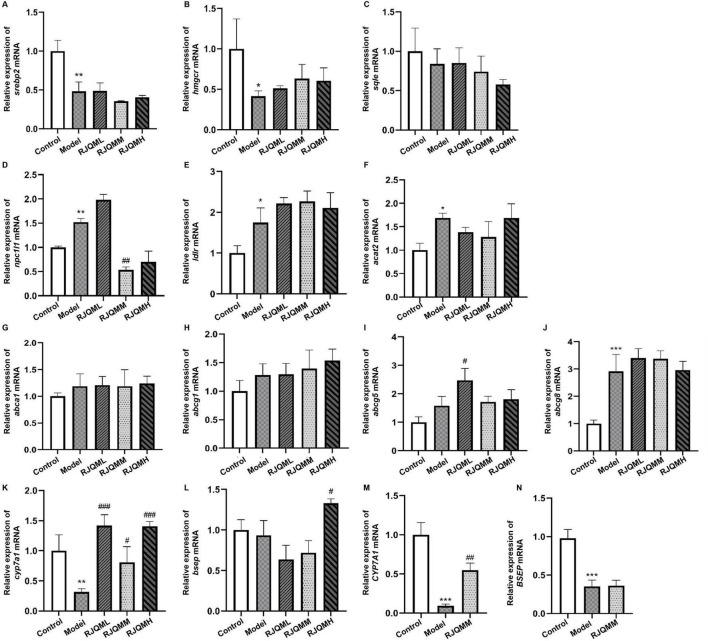
Ruanjian Qingmai granules (RJQM) upregulates *CYP7A1* mRNA expression in zebrafish and cellular models. **(A–L)** Zebrafish atherosclerosis model construction and, quantitative real-time PCR (qRT-PCR) analysis of cholesterol metabolism-related genes after 5 days of RJQM intervention (8∼10 embryos per group, *n* = 3) Control, normal diet; Model, 8% high-cholesterol diet; RJQML, RJQMM, RJQMH, co-treated with 8% high cholesterol diet + 0.025, 0.05, or 0.1 mg/ml/d RJQM, respectively **(M,N)** qRT-PCR detection of *CYP7A1* and *BSEP* mRNA expression in HepaRG cells after 24 h treatment *n* = 3. Control, 10% control serum; Model, 10% control serum + 0.25 mM OA; RJQMM, co-treated with 0.25 mM OA + 10% RJQM drug-containing serum. *p < 0.05 < 0.01, **p < 0.01, ***p < 0.001 compared with the control group; ^#^*p* < 0.05, ^##^*p* < 0.01, ^###^*p* < 0.001 compared with the model group.

Given the central role of cholesterol in lipid metabolism, we initially focused on key regulatory targets involved in four critical processes of cholesterol homeostasis: absorption, synthesis, esterification and efflux (24). Specifically, we investigated the expression levels of the following genes using qRT-PCR: sterol regulatory element binding protein 2 (*SREBP2*), 3-hydroxy-3methylglutaryl-coenzyme A reductase (*HMGCR*), squalene epoxide (*SQLE*), ATP-binding cassette transporters (*ABCA1, ABCG1, ABCG5*, and *ABCG8*), Niemann-Pick C1-Like 1 (*NPC1L1*), low-density lipoprotein receptor (*LDLR*), acyl-coenzyme A cholesterol acyltransferase 2 (*ACAT2*), cholesterol 7α-hydroxylase (*CYP7A1*), and bile salt export pump (*BESP*) (Figures 2A–L).

The results indicated that compared with the control group, *cyp7a1* mRNA expression was significantly downregulated in the model group of zebrafish. After 5 days of intervention with RJQML, RJQMM, and RJQMH, *cyp7a1* mRNA expression level was significantly upregulated in zebrafish (*p* < 0.05).

Consistent with these in vivo findings, our *in vitro* experiments demonstrated that RJQMM significantly upregulated *CYP7A1* mRNA expression levels in HepaRG cells (*p* < 0.05) (Figure 2M), while did not affect the expression level of BESP mRNA (Figure 2N). Therefore, we suggest that RJQM may exert its lipid-lowering effects through *CYP7A1*-mediated regulation of lipid metabolism.

### 3.3 *CYP7A1* inhibitors impair RJQM reprogramming lipid metabolism

**FIGURE 3:**
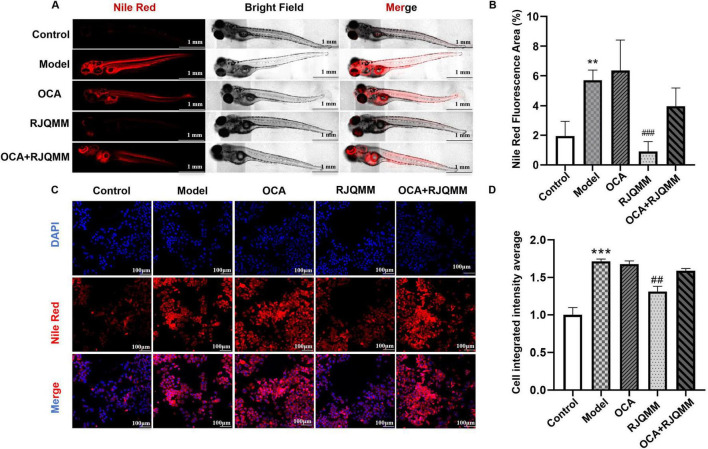
Ruanjian Qingmai granules (RJQM)-mediated lipid metabolism reprogramming is attenuated by *CYP7A1* inhibition. **(A,B)** Nile Red staining **(A)** and fluorescence quantification **(B)** in zebrafish atherosclerosis model after 5 days of RJQM treatment (*n* = 5). Control, normal diet; Model, 8% high-cholesterol diet; RJQMM, co-treated with 8% high cholesterol diet + 0.05 mg/ml/d RJQM; OCA, co-treated with 8% high cholesterol diet + 4 μM of obecholic acid (OCA). OCA + RJQMM, co-treated with 8% high cholesterol diet + 4 μM OCA + 0.05 mg/ml/d RJQM. **(C,D)** Nile Red staining **(C)** and fluorescence quantification **(D)** in HepaRG cells after 24 h of RJQM treatment (*n* = 3). Control, 10% control serum; Model, 10% control serum + 0.25 mM OA; RJQMM, co-treated with 0.25 mM OA + 10% RJQM drug-containing serum; OCA, co-treated with 0.25 mM OA + 30 μM OCA. OCA + RJQMM, co-treated with 0.25 mM OA + 30 μM OCA + 10% RJQM drug-containing serum **p < 0.01, ***p < 0.001 compared with the control group; ^##^*p* < 0.01, ^###^*p* < 0.001 compared with the model group.

To further clarify the role of *CYP7A1* in mediating RJQM’s effects, obecholic acid (OCA), an inhibitor of *CYP7A1*, was used to impede cholesterol efflux (25). As shown in Figures 3A, B, while RJQMM maintained its lipid-reducing effects, these effects were significantly weakened by OCA co-treatment (*p* < 0.05). Consistent with theses *in vivo* findings, the cell experiments (Figure 3C, D) also demonstrated that the effect of RJQMM on reprogramming lipid metabolism was diminished by *CYP7A1* inhibitors (*p* < 0.05). Therefore, we believe that RJQM reprograms lipid metabolism by acting on *CYP7A1*, thus playing a role in the treatment of ASO.

### 3.4 Screening of potential bioactive components of RJQM

**FIGURE 4:**
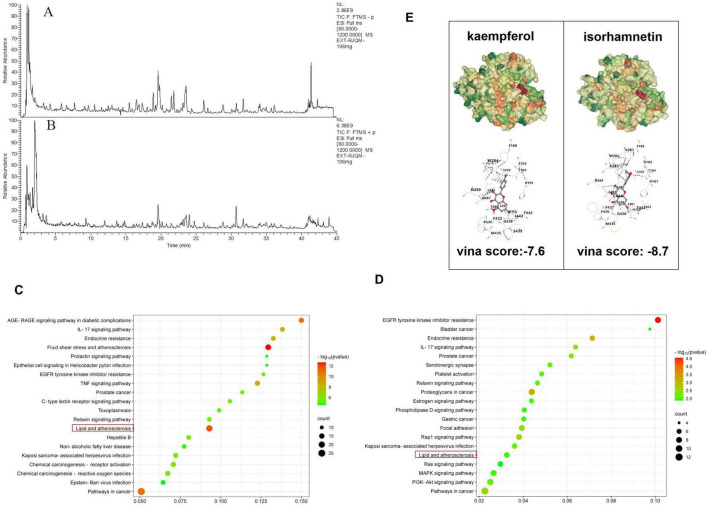
Screening of potential bioactive components of Ruanjian Qingmai granules (RJQM). **(A,B)** LC-MS chromatograms of RJQM in positive ion mode **(A)** and negative ion mode **(B)**. **(C,D)** KEGG pathway enrichment analysis of kaempferol **(C)** and isorhamnetin **(D)**. **(E)** Docking sites and docking fractions of kaempferol, isorhamnetin and *CYP7A1* molecules.

To identify potential bioactive components responsible for RJQM-mediated lipid metabolism reprogramming, we initially screened 20 bioactive components from RJQM using the TCMSP database, applying selection criteria of oral bioavailability ≥ 30% and drug-likeness properties ≥ 0.18 (Supplementary Table 1). Subsequently, serum pharmacochemistry analysis identified 285 chemical components in RJQM extract and 35 hematogenous components in serum (Figures 4A, B and Supplementary Table 2). Among these, kaempferol and isorhamnetin were identified as common components.

To investigate their potential roles in lipid metabolic reprogramming, we predicted the target gene for both compounds and retrieved disease-associated genes from GeneCards and DisGeNET databases, including 2,791 atherosclerosis-related genes, 52 ASO-related genes and 529 hypercholesterolemia-related genes, After removing redundant entries, we performed KEGG pathway enrichment analysis on shared targets. The results suggested that both isorhamnetin and kaempferol may regulate lipid metabolism in atherosclerosis (Figures 4C, D). Molecular docking analysis revealed strong binding interactions between *CYP7A1* and both kaempferol and isorhamnetin, suggesting *CYP7A1* is a potential target of these compounds (Figure 4E). These finding suggest that kaempferol and isorhamnetin are likely the potential active components mediating RJQM’s lipid metabolism reprogramming effects.

### 3.5 Kaempferol and isorhamnetin are the potential bioactive components of RJQM in lipid reprogramming

**FIGURE 5:**
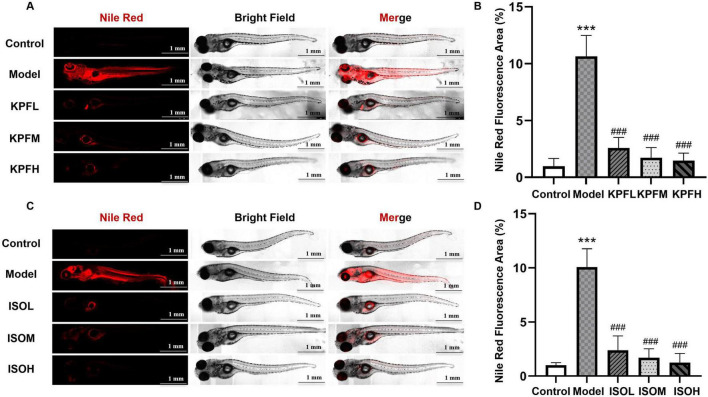
Kaempferol and isorhamnetin reprogrammed lipid metabolism in the zebrafish model. Zebrafish atherosclerosis model was utilized. **(A,B)** Representative Nile Red staining images **(A)** and quantification results **(B)** after 5 days treatment with different doses of kaempferol. **(C,D)** Representative Nile Red staining **(C)** and fluorescence quantification results **(D)** after 5 days treatment with different doses of isorhamnetin. *n* = 5. Control, normal diet; Model, 8% high-cholesterol diet; KPFL, KPFM and KPFH, co-treated with 8% high-cholesterol diet +1, 5, or 10 μg/ml/d kaempferol, respectively; ISOL, ISOM and ISOH, co-treated with 8% high-cholesterol diet +1, 5, or 10 μg/ml/d isorhamnetin, respectively. ***p < 0.001 vs. control group; ^###^*p* < 0.001 vs. model group.

**FIGURE 6:**
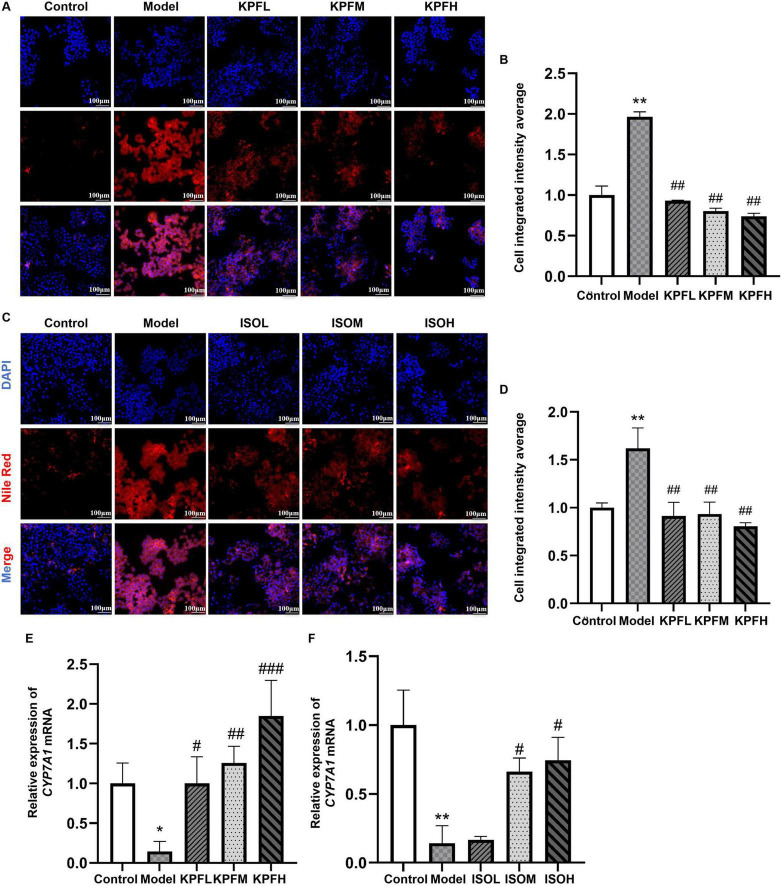
Kaempferol and isorhamnetin reprogrammed lipid metabolism in the cellular model. **(A,B)** Nile Red staining images **(A)** and quantizative fluorescence analysis **(B)** of HepaRG cells treated with different doses of kaempferol for 24 h. **(C,D)** Nile Red staining imgaes **(C)** and fluorescence quantification **(D)** following 24 h treatment with different doses of isorhamnetin. **(E,F**) qRT-PCR analysis of *CYP7A1* mRNA expression levels after 24 h treatment with kaempferol **(E)** or isorhamnetin **(F)**, *n* = 3. Model, HepaRG treated with 0.25 mM OA for 24 h; KPFL, KPFM and KPFH, co-treatment with 0.25 mM OA and kaempferol (5, 10, or 20 μM) for 24 h; ISOL, ISOM and ISOH, co-treatment with 0.25 mM OA and isorhamnetin (5, 10, or 20 μM) for 24 h. Control, give equal volume of solvent. **p < 0.01 vs. control group; ^#^*p* < 0.05, ^##^*p* < 0.01, ^###^*p* < 0.001 compared with the model group.

To further validate whether kaempferol and isorhamnetin exert metabolic reprogramming effects, we re-established a zebrafish atherosclerosis model. The results showed that low, medium, and high doses of kaempferol (KPFL, KPFM, KPFH) significantly reduced lipid deposition in zebrafish (*p* < 0.05) (Figures 5A, B); a similar lipid-lowering effect was observed with different doses of isorhamnetin (ISOL, ISOM, ISOH) (Figures 5C, D) (*p* < 0.05). Subsequently, in the cellular model, we found that compared to the model group, all kaempferol dose groups significantly reduced Nile red fluorescence intensity (Figures 6A, B) (*p* < 0.05); different doses of isorhamnetin also demonstrated lipid-lowering effects (Figures 6C, D) (*p* < 0.05). Notably, KPFL, KPFM, KPFH and ISOM, ISOH significantly upregulated the mRNA expression level of *CYP7A1* in HepaRG cells (*p* < 0.05) (Figures 6E, F). These results confirmed our hypothesis that “kaempferol and isorhamnetin are potential bioactive components through which RJQM mediates lipid reprogramming.”

**FIGURE 7:**
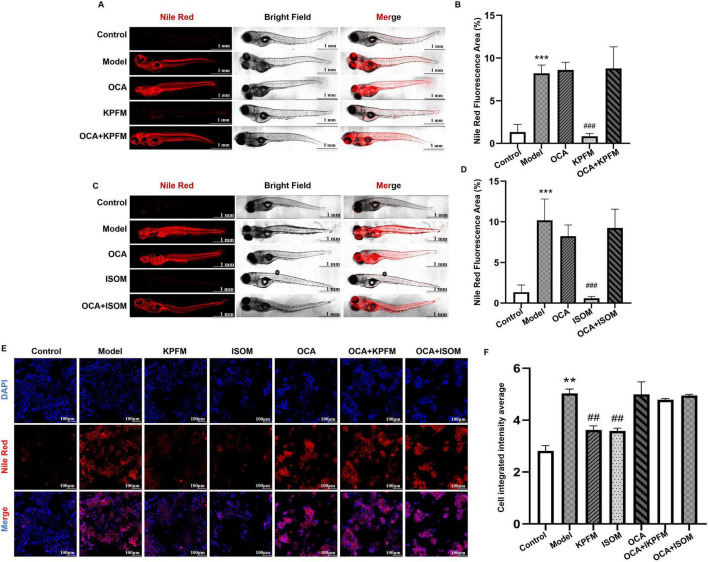
In Zebrafish and cellular models, the reprogramming of lipid metabolism by kaempferol and isorhamnetin was attenuated by *CYP7A1* inhibitors. **(A,B)** and **(C,D)** The Nile Red staining images and fluorescence quantification results at 5 days after kaempferol **(A,B)** or isorhamnetin **(C,D)** intervention, with or without *CYP7A1* inhibitors, in zebrafish atherosclerosis model. Model, 8% high-cholesterol diet; KPFM, co-treated with 8% high-cholesterol diet + 5 μg/ml/d kaempferol; ISOM, co-treated with 8% high-cholesterol diet + 5 μg/ml/d isorhamnetin; OCA, co-treated with 8% high-cholesterol diet +4 μM of OCA. OCA + KPFM and OCA + ISOM, co-treated with 8% high-cholesterol diet + 4 μM OCA + 5 μg/ml/d KPFM or ISOM; Control, was given equal volume of egg water. **(E,F)** Typical Nile Red staining images **(E)** and quantitative results **(F)** of kaempferol or isorhamnetin intervention for 24 h, with or without *CYP7A1* inhibitors, in HepaRG cells. Model, 0.25 mM OA. KPFM and ISOM: co-treated with 0.25 mM OA + 10 μM kaempferol or isorhamnetin, respectively. OCA, co-treated with 0.25 mM OA + 30 μM OCA. OCA + KPFM and OCA + ISOM: co-treated with 0.25 mM OA + 30 μM OCA + 10 μM kaempferol or isorhamnetin. Control, was given equal volume of solvent. **p < 0.01, ***p < 0.001 compared with the control group; ^##^*p* < 0.01, ^###^*p* < 0.001 compared with the model group.

To further clarify whether the metabolic reprogramming effects of kaempferol and isorhamnetin are achieved by regulating *CYP7A1* expression, we employed a *CYP7A1* inhibitor for validation. In the zebrafish model, compared to the model group, KPFM significantly reduced lipid deposition (*p* < 0.05); however, when KPFM was co-administered with OCA, the extent of lipid deposition showed no significant difference from the model group (*p* > 0.05) (Figures 7A, B). Similarly, the lipid-lowering effect of isorhamnetin was attenuated when combined with OCA (*p* > 0.05) (Figures 7C, D). In the cellular model, OCA also diminished the lipid-lowering effects of kaempferol and isorhamnetin (Figures 7E, F). These findings strongly suggest that kaempferol and isorhamnetin are likely potential active components mediating the lipid-lowering effects of RJQM through a *CYP7A1*-dependent mechanism.

## 4 Discussion

Arteriosclerotic obliterans is a type of peripheral vascular disease characterized by the formation of atherosclerotic plaques, leading to the narrowing, hardening, and blockage of the artery lumen. This results in reduced or interrupted distal blood flow (26). ASO is most prevalent among individuals over 65 years of age with peripheral artery disease (27). In China, the number of ASO patients is estimated to reach 45.3 million (28).

Current treatment options for ASO include endovascular angioplasty, endarterectomy, arterial bypass.

Lipids, especially cholesterol, can act as a “catalyst” to promote development of atherosclerosis. Excessive accumulation of cholesterol and the synthesis of cholesterol esters can stimulate macrophages to transform into foam cells, thereby accelerating the formation of atherosclerotic plaques (32, 33). As a result, cholesterol homeostasis plays a vital role in maintaining cellular and bodily functions, making it a key factor in the development of ASO and a central focus of lipid metabolic reprogramming. Furthermore, cholesterol homeostasis holds significant clinical importance in a range of lipid metabolism-related diseases, including cardiovascular diseases (34) and atherosclerosis (35).

Ruanjian Qingmai granules, a classic prescription with well-established clinical efficacy, has been extensively utilized in the treatment of ASO. Our previous studies suggested that RJQM could significantly reduce the active plaque area in ApoE^–/–^ mice (36).This study further confirmed that RJQM effectively reduces lipid deposition at both zebrafish and cellular levels, while also suggesting that *CYP7A1* may serve as a key target of RJQM.

Niemann-Pick C1-Like 1 is a cholesterol transport protein widely expressed in the small intestine and liver. It mediates the uptake of cholesterol into intracellular vesicles through endocytosis, participates in the enterohepatic circulation, and plays a crucial role in maintaining cholesterol metabolic balance (37, 38). In this study, we similarly found that RJQM significantly reduces elevated levels of NPC1L1 mRNA. However, Ezetimibe, which directly targets NPC1L1, has been widely used in the treatment of hypercholesterolemia and atherosclerosis by reducing intestinal cholesterol absorption (39, 40). Therefore, in subsequent studies, we primarily focused on *CYP7A1*.

Cholesterol 7α-hydroxylase, located in the liver endoplasmic reticulum, is the only rate-limiting enzyme in the classical bile acid synthesis pathway and plays an important role in cholesterol efflux (41). Cholesterol is catalyzed by *CYP7A1* and metabolized to 7α-hydroxyl cholesterol, which is subsequently converted into bile acid through a series of reactions (42). The activation of *CYP7A1* promotes the transformation of liver cholesterol into bile acids, thereby reducing cholesterol deposition and realizing lipid metabolic reprogramming (43). *CYP7A1* is primarily regulated by the farnesoid X receptor (FXR), a member of the nuclear receptor superfamily widely expressed in the liver and gastrointestinal tract (44). FXR exerts its biological functions either by inducing its target gene small heterodimer partner (SHP) (45) or by promoting the release of fibroblast growth factor 19 (FGF19, with FGF15 being its murine homolog) (46). Our study, through both in vitro and in vivo experiments, demonstrates that *CYP7A1* likely serves as the key regulatory target through which RJQM reprograms lipid metabolism. However, the deeper mechanisms underlying this process require further investigation.

Bile salt export pump, a member of the ABC superfamily, is primary transporter for bile acid efflux and plays an important role in cholestasis by actively transporting bile acid into the bile duct (47). This study also observed that RJQM up-regulates *BESP* mRNA levels (Figure 4D), suggesting that RJQM may not induce cholestasis while promoting bile acid synthesis and enhancing cholesterol efflux. This further highlights the multi-target and multi-pathway mechanisms characteristic of TCM compounds. However, this hypothesis requires further experimental verification to confirm the absence of adverse effects related to cholestasis during this process.

Furthermore, through integrated analysis of blood component profiling and network pharmacology, we identified two potential bioactive components–kaempferol and isorhamnetin. KEGG pathway analysis revealed that these flavonoids participate in the “Lipid and atherosclerosis” signaling pathway (Figures 4C, D). Molecular docking simulations further indicated favorable binding affinities between both compounds and *CYP7A1*. We thus hypothesize that these flavonoids serve as primary active components through which RJQM exerts lipid metabolic reprogramming effects via targeting *CYP7A1*.

Existing studies have demonstrated the therapeutic efficacy of such flavonoid compounds in cardiovascular disease (48), diabetes (49, 50), cancer (51), while research on their role in ASO remains limited. Therefore, in this study, we we employed both zebrafish and cellular models to validate that these compounds significantly reduce lipid deposition. qRT-PCR results further confirmed their capacity to markedly up-regulate *CYP7A1* mRNA expression. Subsequent experiments using a *CYP7A1* inhibitor demonstrated that the lipid-lowering effects of both compounds were attenuated upon *CYP7A1* inhibition. These findings substantiate our hypothesis that kaempferol and isorhamnetin are the primary active constituents mediating RJQM’s lipid metabolic reprogramming effects through *CYP7A1* modulation, though the underlying mechanisms warrant further investigation.

However, kaempferol exhibits limited oral bioavailability (2%) due to its high first-pass metabolism and rapid metabolic clearance. Despite its low blood-brain barrier permeability, it demonstrates high safety (52), making it a promising candidate for structural modification or optimization to enhance absorption and metabolic stability (53), which represents a key focus for our future research. Similarly, isorhamnetin—another flavonoid compound—shows favorable oral absorption (75%) but also fails to cross the blood-brain barrier. Notably, it may carry potential mutagenic risks (54). Addressing these limitations through drug optimization or multi-drug combination strategies remains a critical challenge for future pharmaceutical development.

## 5 Conclusion

Lipid metabolism reprogramming represents a critical therapeutic approach for the treatment of ASO. *CYP7A1*, a key regulator of bile acid synthesis, plays a pivotal role in promoting cholesterol efflux. Currently, there is a significant gap in the development of lipid-lowering drugs specifically targeting *CYP7A1*. This study not only demonstrates that RJQM promotes cholesterol efflux, reduces lipid deposition, and mitigates the pathogenic factors of ASO but also identifies *CYP7A1* as a key target for RJQM’s lipid metabolism reprogramming effects. Furthermore, the study highlights two primary bioactive components in RJQM-kaempferol and isorhamnetin-which contribute to these therapeutic outcomes. These findings provide a solid experimental foundation for the broader clinical application of RJQM and offer novel insights for development of lipid metabolism reprogramming drugs.

## Data Availability

The original contributions presented in this study are included in this article/Supplementary material, further inquiries can be directed to the corresponding authors.

## Footnotes

1
https://www.tcmsp-e.com/load_intro.php?id=43

2
http://herb.ac.cn/

3
http://swisstargetprediction.ch/

4
http://www.bioinformatics.com.cn/#

5
https://david.ncifcrf.gov/

6
https://www.rcsb.org/

7
https://www.ncbi.nlm.nih.gov/

8
https://cadd.labshare.cn/cb-dock2/php/index.php
