# Supplementary material for: To elucidate the effect of Ruanjian Qingmai granules on arteriosclerosis obliterans from the perspective of cholesterol efflux
Source: Front Med (Lausanne). 2025 Aug 4;12:1510927. doi: 10.3389/fmed.2025.1510927 (PMC12358959; doi:10.3389/fmed.2025.1510927)
Supplement: Supplementary file 1 [file Table_1.docx]

Supplementary Data 1 Network Pharmacological Analysis of RJQM

| **No.** | Mol ID | Molecule Name | MW | OB (%) | DL |
| --- | --- | --- | --- | --- | --- |
| 1 | MOL001792 | DFV | 256.27 | 32.76 | 0.18 |
| 2 | MOL000006 | luteolin | 286.25 | 36.16 | 0.25 |
| 3 | MOL000098 | quercetin | 302.25 | 46.43 | 0.28 |
| 4 | MOL000354 | isorhamnetin | 316.28 | 49.6 | 0.31 |
| 5 | MOL000358 | beta-sitosterol | 414.79 | 36.91 | 0.75 |
| 6 | MOL006115 | kaempferol-3-O-α-L-rhamnosyl(1→2)-β-D-glucoside_qt | 284.28 | 62.87 | 0.24 |
| 7 | MOL001439 | arachidonic acid | 304.52 | 45.57 | 0.2 |
| 8 | MOL001040 | (2R)-5,7-dihydroxy-2-(4-hydroxyphenyl)chroman-4-one | 272.27 | 42.36 | 0.21 |
| 9 | MOL000422 | kaempferol | 286.25 | 41.88 | 0.24 |
| 10 | MOL010578 | N-[(1S)-1-(benzyl)-2-[[(1S)-1-(benzyl)-2-hydroxy-ethyl]amino]-2-keto-ethyl]benzamide | 402.53 | 45.76 | 0.43 |
| 11 | MOL010580 | Diglycol dibenzoate | 314.36 | 59.22 | 0.27 |
| 12 | MOL005440 | Isofucosterol | 412.77 | 43.78 | 0.76 |
| 13 | MOL000296 | Hederagenin | 414.79 | 36.91 | 0.75 |
| 14 | MOL000449 | Stigmasterol | 412.77 | 43.83 | 0.76 |
| 15 | MOL004172 | (1R)-1-[(2S,4aR,4bS,7R,8aS)-7-hydroxy-2,4b,8,8-tetramethyl-4,4a,5,6,7,8a,9,10-octahydro-3H-phenanthren-2-yl]ethane-1,2-diol | 322.54 | 46.7 | 0.31 |
| 16 | MOL004177 | 15alpha-Hydroxy-ent-kaur-16-en-19-oic acid | 318.5 | 58.73 | 0.38 |
| 17 | MOL004179 | Vernolic acid | 296.5 | 37.63 | 0.19 |
| 18 | MOL004180 | Coronaridine | 338.49 | 34.97 | 0.68 |
| 19 | MOL004184 | Siegesesteric acid II | 348.58 | 51.98 | 0.48 |
| 20 | MOL004185 | Siegesmethyletheric acid | 334.55 | 60.72 | 0.43 |

Supplementary Data 2 Serum Pharmacochemistry Analysis of RJQM

| **No.** | **Rt/min** | **Ion mode** | **Measured mass /Da** | **Calculated mass /Da** | **Error /ppm** | **Molecular formula** | **Identification** | **Peak area in the original formula** | **Peak area in serum** |
| --- | --- | --- | --- | --- | --- | --- | --- | --- | --- |
| 1 | 0.61 | [M+H]^+^ | 127.0391 | 127.0390 | 0.663 | C6H6O3 | Methyl 2-furoate/Nicotinic Acid or isomer | 920458361 | 0 |
| 2 | 0.89 | [M-H]^-^ | 181.0710 | 181.0718 | 3.121 | C6H14O6 | D-Mannitol | 964692005 | 0 |
| 3 | 0.90 | [M+H]^+^ | 118.0865 | 118.0863 | 2.026 | C5H11NO2 | Valine | 1376593875 | 0 |
| 4 | 0.94 | [M+H]^+^ | 116.0708 | 116.0706 | 2.080 | C5H9NO2 | Proline | 96291743 | 0 |
| 5 | 0.96 | [M+H]^+^ | 175.1190 | 175.1190 | 0.553 | C6H14N4O2 | L-Arginine | 4864078 | 0 |
| 6 | 1.05 | [M-H]^-^ | 503.1636 | 503.1618 | 3.731 | C18H32O16 | Trisaccharide/Sucrose | 3524721 | 0 |
| 7 | 1.06 | [M+H]^+^ | 132.1020 | 132.1019 | 0.755 | C6H13NO2 | Isoleucine/L-Isoleucine | 52800780 | 0 |
| 8 | 1.09 | [M+H]^+^ | 136.0618 | 136.0618 | -0.031 | C5H5N5 | Adenine | 821251744 | 0 |
| 9 | 1.09 | [M+H]^+^ | 112.0509 | 112.0505 | 2.917 | C4H5N3O | Cytosine | 294678806 | 0 |
| 10 | 1.11 | [M+H]^+^ | 152.0567 | 152.0567 | 0.000 | C5H5N5O | Guanine or isomer | 32122433 | 0 |
| 11 | 1.16 | [M-H]^-^ | 191.0190 | 191.0197 | -3.747 | C6H8O7 | Citric Acid | 23390532358 | 0 |
| 12 | 1.21 | [M+H]^+^ | 169.0356 | 169.0356 | -0.376 | C5H4N4O3 | Uric acid | 43224435 | 0 |
| 13 | 1.28 | [M+H]^+^ | 124.0395 | 124.0393 | 1.305 | C6H5NO2 | Nicotinic Acid | 824866628 | 0 |
| 14 | 1.30 | [M+H]^+^ | 137.0457 | 137.0458 | -0.414 | C5H4N4O | Hypoxanthine or isomer | 288179828 | 0 |
| 15 | 1.34 | [M+H]^+^ | 182.0811 | 182.0812 | -0.381 | C9H11NO3 | Tyrosine | 1139914357 | 0 |
| 16 | 1.34 | [M+H]^+^ | 284.0987 | 284.0989 | -0.071 | C10H13N5O5 | Guanosine or isomer | 79437260 | 0 |
| 17 | 1.40 | [M+H]^+^ | 152.0566 | 152.0567 | -0.702 | C5H5N5O | Guanine or isomer | 19291375 | 0 |
| 18 | 1.44 | [M+H]^+^ | 153.0406 | 153.0407 | -0.543 | C5H4N4O2 | Xanthine or isomer | 40547889 | 0 |
| 19 | 1.46 | [M+H]^+^ | 148.0603 | 148.0604 | -0.786 | C5H9NO4 | Glutamic acid | 18450624 | 0 |
| 20 | 1.55 | [M+H]^+^ | 113.0348 | 113.0346 | 2.156 | C4H4N2O2 | Uracil | 246798940 | 0 |
| 21 | 1.56 | [M-H]^-^ | 243.0622 | 243.0623 | -0.140 | C9H12N2O6 | Uridine | 117626346 | 0 |
| 22 | 1.72 | [M+H]^+^ | 276.1076 | 276.1078 | -0.517 | C11H17NO7 | Sarmentosine | 256058955 | 3544736 |
| 23 | 1.74 | [M+H]^+^ | 156.1382 | 156.1383 | 1.182 | C9H17NO | n-Methylisopelletierine | 8471472797 | 167633814 |
| 24 | 1.84 | [M+H]^+^ | 142.1226 | 142.1226 | -0.021 | C8H15NO | Isopelletierine | 64769018 | 0 |
| 25 | 1.97 | [M+H]^+^ | 132.1020 | 132.1019 | 0.409 | C6H13NO2 | Isoleucine/L-Isoleucine | 62513636 | 0 |
| 26 | 2.03 | [M+H]^+^ | 158.1538 | 158.1539 | 0.815 | C9H19NO | 1-Methyl-2-( β-hydroxypropyl)-piperidine | 95490417397 | 2293249009 |
| 27 | 2.04 | [M-H]^-^ | 169.0135 | 169.0142 | -4.595 | C7H6O5 | Gallic acid | 230317815 | 0 |
| 28 | 2.09 | [M+H]^+^ | 268.1037 | 268.1040 | -0.340 | C10H13N5O4 | Adenosine | 334047859 | 0 |
| 29 | 2.24 | [M+H]^+^ | 152.0567 | 152.0567 | 0.402 | C5H5N5O | Guanine or isomer | 33606080 | 0 |
| 30 | 2.24 | [M+H]^+^ | 137.0457 | 137.0458 | -0.303 | C5H4N4O | Hypoxanthine or isomer | 288179828 | 0 |
| 31 | 2.26 | [M+H]^+^ | 284.0987 | 284.0989 | -0.777 | C10H13N5O5 | Guanosine or isomer | 11961299 | 0 |
| 32 | 3.19 | [M+H]^+^ | 153.0407 | 153.0407 | -0.044 | C5H4N4O2 | Xanthine or isomer | 30665222 | 0 |
| 33 | 5.23 | [M+H]^+^ | 127.0390 | 124.0393 | 0.303 | C6H6O3 | Methyl 2-furoate/Nicotinic Acid or isomer | 298824482 | 0 |
| 34 | 5.43 | [M+H]^+^ | 137.0596 | 137.0597 | -0.105 | C8H8O2 | Methyl benzoate | 192870830 | 9437486 |
| 35 | 5.81 | [M+H]^+^ | 127.0390 | 124.0393 | 0.483 | C6H6O3 | Methyl 2-furoate/Nicotinic Acid or isomer | 520551156 | 0 |
| 36 | 5.83 | [M+H]^+^ | 169.0495 | 169.0495 | -0.127 | C8H8O4 | Vanillic acid | 123321710 | 0 |
| 37 | 6.51 | [M-H]^-^ | 353.0882 | 353.0878 | 1.106 | C16H18O9 | Chlorogenic acid or isomer | 191362530 | 0 |
| 38 | 6.92 | [M-H]^-^ | 181.0498 | 181.0506 | -4.401 | C9H10O4 | Syringaldehyde or isomer | 82070582 | 0 |
| 39 | 7.52 | [M-H]^-^ | 193.0500 | 193.0506 | -3.416 | C10H10O4 | Ferulic acid or isomer | 26096012 | 0 |
| 40 | 7.69 | [M+H]^+^ | 199.0600 | 199.0601 | -0.591 | C9H10O5 | Syringic acid or isomer | 162301910 | 962720 |
| 41 | 7.96 | [M+H]^+^ | 146.0599 | 146.0600 | -0.920 | C9H7NO | Indole-3-Carboxaldehyde | 132296579 | 0 |
| 42 | 8.11 | [M+H]^+^ | 179.0338 | 179.0339 | -0.656 | C9H6O4 | Esculetin or isomer | 26251150 | 0 |
| 43 | 9.12 | [M+H]^+^ | 153.0545 | 153.0546 | -0.132 | C8H8O3 | Vanillin or isomer | 91473843 | 0 |
| 44 | 9.41 | [M-H]^-^ | 177.0185 | 177.0193 | -4.822 | C9H6O4 | Esculetin or isomer | 129335250 | 0 |
| 45 | 9.63 | [M-H]^-^ | 353.0883 | 353.0878 | 1.365 | C16H18O9 | Chlorogenic acid or isomer | 245447937 | 0 |
| 46 | 9.68 | [M-H]^-^ | 193.0499 | 193.0506 | -3.654 | C10H10O4 | Ferulic acid or isomer | 31313911 | 0 |
| 47 | 9.70 | [M-H]^-^ | 179.0341 | 179.0350 | -4.822 | C9H8O4 | Caffeic acid or isomer | 156791667 | 0 |
| 48 | 10.11 | [M-H]^-^ | 343.0827 | 343.0823 | 1.009 | C18H16O7 | 3,7,4'-O-Trimethylquercetin/3,3',4'-Trimethylquercetin or isomer | 25316639 | 0 |
| 49 | 10.52 | [M+H]^+^ | 165.0545 | 165.0546 | -0.982 | C9H8O3 | 4-Hydroxycinnamic acid/Trans-p-hydroxycinnamic acid | 68022601 | 0 |
| 50 | 10.52 | [M+H]^+^ | 147.0439 | 147.0441 | -1.375 | C9H6O2 | Coumarin or isomer | 153854054 | 0 |
| 51 | 10.57 | [M-H]^-^ | 353.0881 | 353.0878 | 0.847 | C16H18O9 | Chlorogenic acid or isomer | 264120704 | 0 |
| 52 | 10.75 | [M-H]^-^ | 503.1608 | 503.1618 | -1.910 | C18H32O16 | Trisaccharide/Sucrose | 4362472 | 0 |
| 53 | 10.83 | [M+H]^+^ | 153.0545 | 153.0546 | -0.147 | C8H8O3 | Vanillin or isomer | 37909292 | 0 |
| 54 | 11.34 | [M-H]^-^ | 181.0499 | 181.0506 | -4.233 | C9H10O4 | Syringaldehyde or isomer | 64665573 | 0 |
| 55 | 11.47 | [M+H]^+^ | 199.0599 | 199.0601 | -0.209 | C9H10O5 | Syringic acid or isomer | 20989511 | 2216553 |
| 56 | 11.47 | [M+H]^+^ | 153.0545 | 153.0546 | -0.162 | C8H8O3 | Vanillin or isomer | 42723388 | 0 |
| 57 | 11.55 | [M+H]^+^ | 147.0439 | 147.0441 | -1.272 | C9H6O2 | Coumarin or isomer | 281062657 | 0 |
| 58 | 12.13 | [M+H]^+^ | 303.0494 | 303.0499 | -1.821 | C15H10O7 | Quercetin/Herbacetin/Pentahydroxyflavone or isomer | 82852885 | 0 |
| 59 | 12.14 | [M+H]^+^ | 465.1025 | 465.1028 | -0.524 | C21H20O12 | Quercetin-3-O-glucoside/Quercetin-3-O-galactoside/Quercetin-3-β-glucopyranoside/Hyperoside or isomer | 21913239 | 0 |
| 60 | 12.21 | [M-H]^-^ | 515.1198 | 515.1195 | 0.607 | C25H24O12 | 3, 4-Dicaffeoylquinic acid or isomer | 81289478 | 0 |
| 61 | 12.40 | [M-H]^-^ | 181.0498 | 181.0506 | -4.486 | C9H10O4 | Syringaldehyde or isomer | 41008313 | 0 |
| 62 | 12.50 | [M-H]^-^ | 193.0500 | 193.0506 | -3.481 | C10H10O4 | Ferulic acid or isomer | 96040980 | 1520696 |
| 63 | 12.90 | [M+H]^+^ | 165.0545 | 165.0546 | -0.520 | C9H8O3 | 4-Hydroxycinnamic acid/Trans-p-hydroxycinnamic acid | 72426671 | 0 |
| 64 | 12.90 | [M+H]^+^ | 147.0439 | 147.0441 | -1.375 | C9H6O2 | Coumarin or isomer | 229269970 | 21311133 |
| 65 | 13.04 | [M-H]^-^ | 179.0341 | 179.0350 | -4.652 | C9H8O4 | Caffeic acid or isomer | 113065741 | 0 |
| 66 | 13.58 | [M-H]^-^ | 281.1396 | 281.1394 | 0.716 | C15H22O5 | 8β,9β-Dihydroxy-1β,10α-epoxy-11β,13-dihydrocostunolide or isomer | 17582933 | 0 |
| 67 | 13.65 | [M+H]^+^ | 387.2007 | 387.2013 | -1.706 | C19H30O8 | Staphylionoside D/Neosedumoside Ⅰ/Neosedumoside Ⅱ or isomer | 27888942 | 0 |
| 68 | 13.87 | [M-H]^-^ | 193.0500 | 193.0506 | -3.495 | C10H10O4 | Ferulic acid or isomer | 58814350 | 0 |
| 69 | 13.95 | [M+H]^+^ | 287.0545 | 287.0550 | -1.671 | C15H10O6 | Luteolin/Kaempferol or isomer | 43680062 | 0 |
| 70 | 13.97 | [M+H]^+^ | 611.1600 | 611.1607 | -1.026 | C27H30O16 | Quercetin-3-O-neohesperidoside/Rutin or isomer | 158439015 | 0 |
| 71 | 14.01 | [M+H]^+^ | 211.1439 | 211.1441 | -0.941 | C11H18N2O2 | (3S, 8aS)-3-(R-sec-Butyl)-hexahydropyrrolo [1, 2a] pyrazine-1, 4-dione/(3S, 8aS)-3-(S-sec-Butyl)-hexahydropyrrolo [1, 2a] pyrazine-1, 4-dione | 36694825 | 0 |
| 72 | 14.24 | [M+H]^+^ | 465.1024 | 465.1028 | -0.787 | C21H20O12 | Quercetin-3-O-glucoside/Quercetin-3-O-galactoside/Quercetin-3-β-glucopyranoside/Hyperoside or isomer | 14998505 | 0 |
| 73 | 14.34 | [M+H]^+^ | 393.2478 | 393.2483 | -1.173 | C19H36O8 | Sedumoside A1/Sedumoside A2/Sedumoside A3/Sedumoside B | 10365646 | 0 |
| 74 | 14.34 | [M+H]^+^ | 375.2373 | 375.2377 | -1.214 | C19H34O7 | Sedumoside F1/Myrsinionoside A or isomer | 7287294 | 0 |
| 75 | 14.56 | [M-H]^-^ | 593.1519 | 593.1512 | 1.116 | C27H30O15 | Kaempferol-3- O-neohesperidoside or isomer | 22154514 | 0 |
| 76 | 14.57 | [M+H]^+^ | 641.1703 | 641.1712 | -1.367 | C28H32O17 | Isorhamnetin-3,7-diglucoside | 634272038 | 0 |
| 77 | 14.57 | [M+H]^+^ | 611.1600 | 611.1607 | -1.026 | C27H30O16 | Quercetin-3-O-neohesperidoside/Rutin or isomer | 41803033 | 0 |
| 78 | 14.57 | [M+H]^+^ | 479.1182 | 479.1184 | -0.498 | C22H22O12 | Isorhamnetin-7-glucoside/Isorhamnetin-3-β-glucoside/Isorhamnetin-3-O-glucoside/Isorhamnetin-3-O-β-galactoside or isomer | 44715259 | 0 |
| 79 | 14.57 | [M+H]^+^ | 317.0651 | 317.0656 | -1.627 | C16H12O7 | 3-Methylquercetin/Isorhamnetin/Isorhamnetin/Rhamnetin or isomer | 173299703 | 0 |
| 80 | 14.78 | [M+H]^+^ | 211.1438 | 211.1441 | -1.375 | C11H18N2O2 | (3S, 8aS)-3-(R-sec-Butyl)-hexahydropyrrolo [1, 2a] pyrazine-1, 4-dione/(3S, 8aS)-3-(S-sec-Butyl)-hexahydropyrrolo [1, 2a] pyrazine-1, 4-dione | 84786432 | 0 |
| 81 | 15.07 | [M+H]^+^ | 611.1600 | 611.1607 | -0.627 | C27H30O16 | Quercetin-3-O-neohesperidoside/Rutin or isomer | 15666548 | 0 |
| 82 | 15.48 | [M+H]^+^ | 147.0438 | 147.0441 | -1.479 | C9H6O2 | Coumarin or isomer | 311514255 | 0 |
| 83 | 15.90 | [M+H]^+^ | 671.1811 | 671.1818 | -1.042 | C29H34O18 | Limoeitrin-3,7-diglueoside | 175176086 | 0 |
| 84 | 15.90 | [M+H]^+^ | 509.1282 | 509.1290 | -1.438 | C23H24O13 | Limocitrin-3-glucoside or isomer | 10744230 | 0 |
| 85 | 15.90 | [M+H]^+^ | 347.0757 | 347.0761 | -1.238 | C17H14O8 | Limocitrin or isomer | 61482080 | 0 |
| 86 | 15.93 | [M-H]^-^ | 463.0890 | 463.0882 | 1.706 | C21H20O12 | Quercetin-3-O-glucoside/Quercetin-3-O-galactoside/Quercetin-3-β-glucopyranoside/Hyperoside or isomer | 177104911 | 0 |
| 87 | 16.23 | [M+H]^+^ | 387.2009 | 387.2013 | -1.234 | C19H30O8 | Staphylionoside D/Neosedumoside Ⅰ/Neosedumoside Ⅱ or isomer | 123207080 | 0 |
| 88 | 16.44 | [M+H]^+^ | 375.2371 | 375.2377 | -1.784 | C19H34O7 | Sedumoside F1/Myrsinionoside A or isomer | 4418032 | 0 |
| 89 | 16.46 | [M+H]^+^ | 393.2475 | 393.2483 | -1.949 | C19H36O8 | Sedumoside A1/Sedumoside A2/Sedumoside A3/Sedumoside B | 5887495 | 0 |
| 90 | 16.52 | [M+H]^+^ | 387.2008 | 387.2013 | -1.391 | C19H30O8 | Staphylionoside D/Neosedumoside Ⅰ/Neosedumoside Ⅱ or isomer | 30816636 | 0 |
| 91 | 16.53 | [M-H]^-^ | 381.2192 | 389.2181 | 2.869 | C19H34O8 | Sedumoside C/Sedumoside D/Alangionoside A | 8248882 | 0 |
| 92 | 16.55 | [M-H]^-^ | 387.2030 | 387.2024 | 1.372 | C19H32O8 | Sedumoside H/Sedumoside I/3-Hydroxy-5,6-epoxy-β-ionol 9-O-β-D-glucopyranoside/Neosedumoside Ⅳ | 69278596 | 0 |
| 93 | 16.71 | [M+H]^+^ | 257.0803 | 257.0808 | -1.996 | C15H12O4 | Liquiritigenin/Isoliquiritigenin or isomer | 40054724 | 0 |
| 94 | 16.72 | [M-H]^-^ | 417.1197 | 417.1191 | 1.401 | C21H22O9 | Liquiritin/Isoliquiritin | 36699216 | 0 |
| 95 | 16.76 | [M-H]^-^ | 193.0499 | 193.0506 | -3.654 | C10H10O4 | Ferulic acid or isomer | 452944903 | 0 |
| 96 | 16.83 | [M+H]^+^ | 387.2007 | 387.2013 | -1.628 | C19H30O8 | Staphylionoside D/Neosedumoside Ⅰ/Neosedumoside Ⅱ or isomer | 174874228 | 0 |
| 97 | 16.86 | [M-H]^-^ | 367.1766 | 367.1762 | 0.949 | C19H28O7 | Neosedumoside Ⅲ or isomer | 51113062 | 0 |
| 98 | 16.86 | [M-H]^-^ | 331.0825 | 331.0823 | 0.492 | C17H16O7 | 5,4'-Dihydroxy-7,3'-dimethoxyflavononol | 13874761 | 0 |
| 99 | 16.88 | [M+H]^+^ | 375.2371 | 375.2377 | -1.702 | C19H34O7 | Sedumoside F1/Myrsinionoside A or isomer | 22215079 | 0 |
| 100 | 16.90 | [M+H]^+^ | 393.2477 | 393.2483 | -1.483 | C19H36O8 | Sedumoside A1/Sedumoside A2/Sedumoside A3/Sedumoside B | 27257120 | 0 |
| 101 | 16.98 | [M+H]^+^ | 683.1808 | 683.1818 | -1.381 | C30H34O18 | Sarmenoside Ⅴ/Sarmenoside Ⅵ or isomer | 14438742 | 0 |
| 102 | 17.26 | [M-H]^-^ | 463.0888 | 463.0882 | 1.377 | C21H20O12 | Quercetin-3-O-glucoside/Quercetin-3-O-galactoside/Quercetin-3-β-glucopyranoside/Hyperoside or isomer | 41412718 | 0 |
| 103 | 17.34 | [M-H]^-^ | 389.2184 | 389.2181 | 0.830 | C19H34O8 | Sedumoside C/Sedumoside D/Alangionoside A | 51807871 | 0 |
| 104 | 17.40 | [M+H]^+^ | 257.0804 | 257.0808 | -1.877 | C15H12O4 | Liquiritigenin/Isoliquiritigenin or isomer | 24073456 | 0 |
| 105 | 17.40 | [M+H]^+^ | 185.1535 | 185.1536 | -0.804 | C11H20O2 | 5-Heptyldihydro-3-furanone or isomer | 12439280 | 0 |
| 106 | 17.47 | [M-H]^-^ | 243.1600 | 243.1602 | -0.601 | C13H24O4 | Sarmentoic acid or isomer | 264220472 | 3378614 |
| 107 | 18.04 | [M+H]^+^ | 389.2164 | 389.2170 | -1.448 | C19H32O8 | Sedumoside H/Sedumoside I/3-Hydroxy-5,6-epoxy-β-ionol 9-O-β-D-glucopyranoside/Neosedumoside Ⅳ | 203206351 | 0 |
| 108 | 18.47 | [M-H]^-^ | 447.0935 | 447.0922 | 3.016 | C21H20O11 | Orientin/Luteolin-7-glucoside/Quercitrin/Kaempferol-7-O-β-D-glucopyranoside/Quercetin 3-O-L-rhamnoside | 395785541 | 0 |
| 109 | 18.47 | [M+H]^+^ | 287.0542 | 287.0550 | -2.947 | C15H10O6 | Luteolin/Kaempferol or isomer | 56423140 | 0 |
| 110 | 18.55 | [M-H]^-^ | 181.0498 | 181.0506 | -4.823 | C9H10O4 | Syringaldehyde or isomer | 87507041 | 11335336 |
| 111 | 18.57 | [M-H]^-^ | 739.2104 | 739.2091 | 1.823 | C33H40O19 | Grosvenorine/Kaempferol-3-O-( 2G -α-L- rhamnosyl) -rutoside | 19986478 | 0 |
| 112 | 18.67 | [M-H]^-^ | 515.1198 | 515.1195 | 0.489 | C25H24O12 | 3, 4-Dicaffeoylquinic acid or isomer | 170081265 | 0 |
| 113 | 18.80 | [M-H]^-^ | 463.0888 | 463.0882 | 1.377 | C21H20O12 | Quercetin-3-O-glucoside/Quercetin-3-O-galactoside/Quercetin-3-β-glucopyranoside/Hyperoside or isomer | 19681935 | 0 |
| 114 | 18.90 | [M-H]^-^ | 769.2205 | 769.2197 | 1.030 | C34H42O20 | Sarmenoside Ⅳ/Typhaneoside | 209309169 | 0 |
| 115 | 18.90 | [M-H]^-^ | 483.2963 | 483.2963 | 0.409 | C26H44O8 | Darutoside/Pubeside A/Pubeside B/Siegesbeckioside | 349132777 | 0 |
| 116 | 18.90 | [M-H]^-^ | 481.2812 | 481.2807 | 1.340 | C26H42O8 | Ent-2-oxo-15,16-dihydroxypimar-8(14)-en-16-O-β-glucopyranoside | 29290145 | 0 |
| 117 | 18.91 | [M+H]^+^ | 625.1755 | 625.1763 | -1.238 | C28H32O16 | Isorhamnetin-3- O-neohesperidoside/Isorhamnetin-3-O-rutoside or isomer | 28846026 | 0 |
| 118 | 18.91 | [M+H]^+^ | 317.0651 | 317.0656 | -1.530 | C16H12O7 | 3-Methylquercetin/Isorhamnetin/Isorhamnetin/Rhamnetin or isomer | 84234258 | 0 |
| 119 | 18.91 | [M+H]^+^ | 287.0545 | 287.0550 | -1.777 | C15H10O6 | Luteolin/Kaempferol or isomer | 26993546 | 0 |
| 120 | 18.92 | [M-H]^-^ | 593.1518 | 593.1512 | 1.014 | C27H30O15 | Kaempferol-3- O-neohesperidoside or isomer | 38186480 | 0 |
| 121 | 19.06 | [M+H]^+^ | 479.1179 | 479.1184 | -1.135 | C22H22O12 | Isorhamnetin-7-glucoside/Isorhamnetin-3-β-glucoside/Isorhamnetin-3-O-glucoside/Isorhamnetin-3-O-β-galactoside or isomer | 161331509 | 0 |
| 122 | 19.21 | [M-H]^-^ | 755.2049 | 755.2040 | 1.163 | C33H40O20 | Quercetin-3-O-( 2G -α-L- rhamnosyl) -rutoside | 764277768 | 0 |
| 123 | 19.21 | [M+H]^+^ | 449.1070 | 449.1078 | -4.252 | C21H20O11 | Orientin/Luteolin-7-glucoside/Quercitrin/Kaempferol-7-O-β-D-glucopyranoside/Quercetin 3-O-L-rhamnoside | 390141877 | 0 |
| 124 | 19.21 | [M+H]^+^ | 317.0651 | 317.0656 | -1.627 | C16H12O7 | 3-Methylquercetin/Isorhamnetin/Isorhamnetin/Rhamnetin or isomer | 96089139 | 59205 |
| 125 | 19.21 | [M+H]^+^ | 303.0492 | 303.0499 | -2.324 | C15H10O7 | Quercetin/Herbacetin/Pentahydroxyflavone or isomer | 189213597 | 0 |
| 126 | 19.21 | [M+H]^+^ | 287.0543 | 287.0550 | -2.415 | C15H10O6 | Luteolin/Kaempferol or isomer | 13399479 | 0 |
| 127 | 19.22 | [M-H]^-^ | 623.1622 | 623.1618 | 0.760 | C28H32O16 | Isorhamnetin-3- O-neohesperidoside/Isorhamnetin-3-O-rutoside or isomer | 121989746 | 0 |
| 128 | 19.22 | [M-H]^-^ | 593.1518 | 593.1512 | 1.014 | C27H30O15 | Kaempferol-3- O-neohesperidoside or isomer | 65221991 | 0 |
| 129 | 19.29 | [M+H]^+^ | 285.1118 | 285.1121 | -1.226 | C17H16O4 | 3,4-Dimethoxy-2',4'-dihydroxychalcone | 12794068 | 0 |
| 130 | 19.55 | [M-H]^-^ | 917.2366 | 917.2357 | 1.006 | C42H46O23 | Sarmenoside Ⅲ | 551444949 | 0 |
| 131 | 19.56 | [M+H]^+^ | 773.1895 | 773.1924 | -3.753 | C36H36O19 | Quercetin 3-O-α-( 6'"-p-coumaroylglucosyl-β-1,2-rhamnoside) or isomer | 6973884 | 0 |
| 132 | 19.56 | [M+H]^+^ | 449.1072 | 449.1078 | -3.844 | C21H20O11 | Orientin/Luteolin-7-glucoside/Quercitrin/Kaempferol-7-O-β-D-glucopyranoside/Quercetin 3-O-L-rhamnoside | 74401481 | 0 |
| 133 | 19.56 | [M+H]^+^ | 303.0493 | 303.0499 | -2.223 | C15H10O7 | Quercetin/Herbacetin/Pentahydroxyflavone or isomer | 8053927 | 0 |
| 134 | 19.60 | [M+H]^+^ | 241.1430 | 241.1434 | -1.784 | C13H20O4 | (6S, 9R)-2-Hydroxy-4-(2, 6, 6-trimethyl-4-oxo-cyclohex-2-enyl)-butyric acid | 7425800350 | 25367999 |
| 135 | 19.64 | [M+H]^+^ | 625.1754 | 625.1763 | -1.531 | C28H32O16 | Isorhamnetin-3- O-neohesperidoside/Isorhamnetin-3-O-rutoside or isomer | 22556570 | 0 |
| 136 | 19.77 | [M+H]^+^ | 191.1064 | 191.1067 | -1.583 | C12H14O2 | Ethyl 4-methylcinnamate or isomer | 30202446 | 0 |
| 137 | 19.89 | [M-H]^-^ | 463.0887 | 463.0882 | 1.179 | C21H20O12 | Quercetin-3-O-glucoside/Quercetin-3-O-galactoside/Quercetin-3-β-glucopyranoside/Hyperoside or isomer | 59321881 | 0 |
| 138 | 20.11 | [M-H]^-^ | 681.1688 | 681.1672 | 2.239 | C30H34O18 | Sarmenoside Ⅴ/Sarmenoside Ⅵ or isomer | 5083685 | 0 |
| 139 | 20.17 | [M+H]^+^ | 369.1902 | 369.1908 | -1.444 | C19H28O7 | Neosedumoside Ⅲ or isomer | 1784462911 | 0 |
| 140 | 20.22 | [M-H]^-^ | 345.0618 | 345.0616 | 0.513 | C17H14O8 | Limocitrin or isomer | 10722881 | 0 |
| 141 | 20.35 | [M-H]^-^ | 403.1404 | 403.1398 | 1.415 | C21H24O8 | Orientalide or isomer | 8792177 | 0 |
| 142 | 20.60 | [M+H]^+^ | 433.1125 | 433.1129 | -1.076 | C21H20O10 | Apigenin-7-O-β-D-glucopyranoside or isomer | 61941293 | 0 |
| 143 | 20.60 | [M+H]^+^ | 271.0593 | 271.0601 | -2.855 | C15H10O5 | 7,3',4'-Trimethoxy luteolin/7,3',4'-Trihydroxyflavone/Apigenin or isomer | 10948546 | 0 |
| 144 | 20.63 | [M-H]^-^ | 681.1685 | 681.1672 | 1.791 | C30H34O18 | Sarmenoside Ⅴ/Sarmenoside Ⅵ or isomer | 3906427 | 0 |
| 145 | 20.79 | [M+H]^+^ | 331.0804 | 331.0812 | -2.371 | C17H14O7 | 3,7-Dimethylquercetin/3,4'-O-Dimethylquercetin/3',4'-Dimethoxy quercetin/3,3'-Dimethoxy quercetin/Tricin | 11995653 | 0 |
| 146 | 20.84 | [M-H]^-^ | 609.1467 | 609.1461 | 0.919 | C27H30O16 | Quercetin-3-O-neohesperidoside/Rutin or isomer | 34567068 | 0 |
| 147 | 20.91 | [M+H]^+^ | 479.1179 | 479.1184 | -1.007 | C22H22O12 | Isorhamnetin-7-glucoside/Isorhamnetin-3-β-glucoside/Isorhamnetin-3-O-glucoside/Isorhamnetin-3-O-β-galactoside or isomer | 63269492 | 0 |
| 148 | 20.99 | [M+H]^+^ | 287.0544 | 287.0550 | -2.096 | C15H10O6 | Luteolin/Kaempferol or isomer | 24476911 | 0 |
| 149 | 21.00 | [M-H]^-^ | 739.2104 | 739.2091 | 1.740 | C33H40O19 | Grosvenorine/Kaempferol-3-O-( 2G -α-L- rhamnosyl) -rutoside | 82626250 | 0 |
| 150 | 21.00 | [M+H]^+^ | 433.1124 | 433.1129 | -1.287 | C21H20O10 | Apigenin-7-O-β-D-glucopyranoside or isomer | 48119066 | 0 |
| 151 | 21.03 | [M-H]^-^ | 901.2421 | 901.2408 | 1.476 | C42H46O22 | Sarmenoside Ⅰ/Sarmenoside Ⅱ | 166671297 | 0 |
| 152 | 21.05 | [M-H]^-^ | 771.1790 | 771.1778 | 1.575 | C36H36O19 | Quercetin 3-O-α-( 6'"-p-coumaroylglucosyl-β-1,2-rhamnoside) or isomer | 14598625 | 0 |
| 153 | 21.17 | [M-H]^-^ | 403.1407 | 403.1398 | 2.096 | C21H24O8 | Orientalide or isomer | 5819569 | 0 |
| 154 | 21.24 | [M+H]^+^ | 301.0697 | 301.0707 | -3.197 | C16H12O6 | Kaempferide or isomer | 12931594 | 0 |
| 155 | 21.25 | [M-H]^-^ | 515.1198 | 515.1195 | 0.607 | C25H24O12 | 3, 4-Dicaffeoylquinic acid or isomer | 195338267 | 0 |
| 156 | 21.38 | [M-H]^-^ | 281.1395 | 281.1394 | 0.282 | C15H22O5 | 8β,9β-Dihydroxy-1β,10α-epoxy-11β,13-dihydrocostunolide or isomer | 23906552 | 0 |
| 157 | 21.40 | [M-H]^-^ | 901.2422 | 901.2408 | 1.544 | C42H46O22 | Sarmenoside Ⅰ/Sarmenoside Ⅱ | 61825106 | 0 |
| 158 | 21.46 | [M+H]^+^ | 493.1334 | 493.1341 | -1.401 | C23H24O12 | Tricin-7-O-β-D-glucoside | 328386746 | 0 |
| 159 | 21.47 | [M+H]^+^ | 331.0803 | 331.0812 | -2.832 | C17H14O7 | 3,7-Dimethylquercetin/3,4'-O-Dimethylquercetin/3',4'-Dimethoxy quercetin/3,3'-Dimethoxy quercetin/Tricin | 43139801 | 0 |
| 160 | 21.48 | [M-H]^-^ | 507.1144 | 507.1144 | -0.007 | C23H24O13 | Limocitrin-3-glucoside or isomer | 62383303 | 0 |
| 161 | 21.54 | [M+H]^+^ | 433.1122 | 433.1129 | -1.569 | C21H20O10 | Apigenin-7-O-β-D-glucopyranoside or isomer | 61086763 | 0 |
| 162 | 21.54 | [M+H]^+^ | 287.0544 | 287.0550 | -2.202 | C15H10O6 | Luteolin/Kaempferol or isomer | 36240333 | 0 |
| 163 | 21.56 | [M+H]^+^ | 257.0803 | 257.0808 | -2.233 | C15H12O4 | Liquiritigenin/Isoliquiritigenin or isomer | 6861756 | 0 |
| 164 | 21.71 | [M-H]^-^ | 553.2872 | 553.2866 | 1.095 | C25H46O13 | Sedumoside A5 or isomer | 8925615 | 0 |
| 165 | 21.71 | [M-H]^-^ | 477.1041 | 477.1038 | 0.516 | C22H22O12 | Isorhamnetin-7-glucoside/Isorhamnetin-3-β-glucoside/Isorhamnetin-3-O-glucoside/Isorhamnetin-3-O-β-galactoside or isomer | 34945550 | 0 |
| 166 | 21.82 | [M-H]^-^ | 333.1700 | 333.1707 | -2.385 | C19H26O5 | 8β-Isobutyryloxy-1β,10α-epoxycostunolide/9β-Hydroxy-8β-isobutyryloxycostunolide/14-Hydroxy-8β-isobutyryloxycostunolide/9β-Hydroxy-8β-methacryloyloxycostunolide | 6654080 | 0 |
| 167 | 21.82 | [M-H]^-^ | 243.1600 | 243.1602 | -0.924 | C13H24O4 | Sarmentoic acid or isomer | 3131627979 | 13721648 |
| 168 | 21.92 | [M-H]^-^ | 623.1625 | 623.1618 | 1.250 | C28H32O16 | Isorhamnetin-3- O-neohesperidoside/Isorhamnetin-3-O-rutoside or isomer | 16465979 | 0 |
| 169 | 22.00 | [M-H]^-^ | 417.1197 | 417.1191 | 1.328 | C21H22O9 | Liquiritin/Isoliquiritin | 13157013 | 0 |
| 170 | 22.09 | [M-H]^-^ | 301.0356 | 301.0354 | 0.893 | C15H10O7 | Quercetin/Herbacetin/Pentahydroxyflavone or isomer | 16078643 | 0 |
| 171 | 22.13 | [M+H]^+^ | 509.1282 | 509.1290 | -1.438 | C23H24O13 | Limocitrin-3-glucoside or isomer | 38718526 | 0 |
| 172 | 22.24 | [M+H]^+^ | 377.2528 | 377.2534 | -1.598 | C19H36O7 | Myrsinionoside D/Alangionoside J or isomer | 11002576 | 0 |
| 173 | 22.29 | [M-H]^-^ | 477.1043 | 477.1038 | 0.900 | C22H22O12 | Isorhamnetin-7-glucoside/Isorhamnetin-3-β-glucoside/Isorhamnetin-3-O-glucoside/Isorhamnetin-3-O-β-galactoside or isomer | 14417639 | 0 |
| 174 | 22.69 | [M-H]^-^ | 477.1041 | 477.1038 | 0.452 | C22H22O12 | Isorhamnetin-7-glucoside/Isorhamnetin-3-β-glucoside/Isorhamnetin-3-O-glucoside/Isorhamnetin-3-O-β-galactoside or isomer | 32231935 | 0 |
| 175 | 22.74 | [M+H]^+^ | 389.2162 | 389.2170 | -1.997 | C19H32O8 | Sedumoside H/Sedumoside I/3-Hydroxy-5,6-epoxy-β-ionol 9-O-β-D-glucopyranoside/Neosedumoside Ⅳ | 56864970 | 0 |
| 176 | 22.93 | [M+H]^+^ | 191.1064 | 191.1067 | -1.184 | C12H14O2 | Ethyl 4-methylcinnamate or isomer | 47885957 | 0 |
| 177 | 22.96 | [M-H]^-^ | 755.1843 | 755.1829 | 1.905 | C36H36O18 | Quercetin-3-O-α-( 6'"-caffeoylglucosyl-β-1,2-rhamnoside) | 5990963 | 0 |
| 178 | 23.10 | [M+H]^+^ | 375.2370 | 375.2377 | -2.028 | C19H34O7 | Sedumoside F1/Myrsinionoside A or isomer | 3982265 | 0 |
| 179 | 23.13 | [M+H]^+^ | 447.0915 | 447.0922 | -1.557 | C21H18O11 | Baicalin | 1971852751 | 4966396 |
| 180 | 23.13 | [M+H]^+^ | 271.0592 | 271.0601 | -3.305 | C15H10O5 | 7,3',4'-Trimethoxy luteolin/7,3',4'-Trihydroxyflavone/Apigenin or isomer | 231568449 | 0 |
| 181 | 23.14 | [M+H]^+^ | 287.0546 | 287.0550 | -1.565 | C15H10O6 | Luteolin/Kaempferol or isomer | 16013728 | 0 |
| 182 | 23.15 | [M+H]^+^ | 523.3103 | 523.3113 | -1.884 | C25H46O11 | Sedumoside E1/Sedumoside E2 | 2371129 | 0 |
| 183 | 23.23 | [M-H]^-^ | 553.2870 | 553.2866 | 0.764 | C25H46O13 | Sedumoside A5 or isomer | 37007241 | 0 |
| 184 | 23.32 | [M+H]^+^ | 377.2528 | 377.2534 | -1.598 | C19H36O7 | Myrsinionoside D/Alangionoside J or isomer | 20090382 | 0 |
| 185 | 23.41 | [M+H]^+^ | 433.1122 | 433.1129 | -1.710 | C21H20O10 | Apigenin-7-O-β-D-glucopyranoside or isomer | 21499546 | 0 |
| 186 | 23.43 | [M+H]^+^ | 191.1064 | 191.1067 | -1.413 | C12H14O2 | Ethyl 4-methylcinnamate or isomer | 192253673 | 1679596 |
| 187 | 23.69 | [M-H]^-^ | 507.1147 | 507.1144 | 0.535 | C23H24O13 | Limocitrin-3-glucoside or isomer | 4591895 | 0 |
| 188 | 23.93 | [M+H]^+^ | 191.1064 | 191.1067 | -1.184 | C12H14O2 | Ethyl 4-methylcinnamate or isomer | 19094821 | 0 |
| 189 | 24.08 | [M-H]^-^ | 351.2180 | 351.2177 | 0.999 | C20H32O5 | Ent-2β,15,16-trihydroxypimar-8(14)-en-19-oic acid/Ent-16β,17,18-trihydroxy-kauran-19-oic acid | 185928832 | 1206915 |
| 190 | 24.34 | [M+H]^+^ | 283.1535 | 283.1540 | -1.647 | C15H22O5 | 8β,9β-Dihydroxy-1β,10α-epoxy-11β,13-dihydrocostunolide or isomer | 90208401 | 0 |
| 191 | 24.36 | [M+H]^+^ | 523.3107 | 523.3113 | -1.068 | C25H46O11 | Sedumoside E1/Sedumoside E2 | 26495707 | 0 |
| 192 | 24.36 | [M+H]^+^ | 377.2528 | 377.2534 | -1.517 | C19H36O7 | Myrsinionoside D/Alangionoside J or isomer | 4377571 | 0 |
| 193 | 24.63 | [M+H]^+^ | 285.0753 | 285.0758 | -1.625 | C16H12O5 | Glycitein or isomer | 16719041 | 497041 |
| 194 | 24.66 | [M+H]^+^ | 271.0591 | 271.0601 | -3.868 | C15H10O5 | 7,3',4'-Trimethoxy luteolin/7,3',4'-Trihydroxyflavone/Apigenin or isomer | 10681694 | 0 |
| 195 | 24.98 | [M-H]^-^ | 519.2813 | 519.2811 | 0.435 | C25H44O11 | Sedumoside F2/Sedumoside G or isomer | 10087398 | 0 |
| 196 | 25.05 | [M+H]^+^ | 287.0545 | 287.0550 | -1.671 | C15H10O6 | Luteolin/Kaempferol or isomer | 21294506 | 640713 |
| 197 | 25.06 | [M-H]^-^ | 285.0406 | 285.0405 | 0.660 | C15H10O6 | Luteolin/Kaempferol or isomer | 33059323 | 647318 |
| 198 | 25.69 | [M+H]^+^ | 285.0753 | 285.0758 | -1.518 | C16H12O5 | Glycitein or isomer | 13560230 | 552777 |
| 199 | 25.87 | [M-H]^-^ | 519.2814 | 519.2811 | 0.552 | C25H44O11 | Sedumoside F2/Sedumoside G or isomer | 202324868 | 0 |
| 200 | 26.00 | [M+H]^+^ | 285.0749 | 285.0758 | -3.016 | C16H12O5 | Glycitein or isomer | 24264883 | 174131 |
| 201 | 26.05 | [M-H]^-^ | 315.0513 | 315.0510 | 0.773 | C16H12O7 | 3-Methylquercetin/Isorhamnetin/Isorhamnetin/Rhamnetin or isomer | 14333816 | 611501 |
| 202 | 26.56 | [M-H]^-^ | 315.0514 | 315.0510 | 0.258 | C16H12O7 | 3-Methylquercetin/Isorhamnetin/Isorhamnetin/Rhamnetin or isomer | 10763798 | 0 |
| 203 | 26.65 | [M+H]^+^ | 285.0749 | 285.0758 | -3.016 | C16H12O5 | Glycitein or isomer | 65097299 | 812417 |
| 204 | 26.84 | [M-H]^-^ | 519.2813 | 519.2811 | 0.317 | C25H44O11 | Sedumoside F2/Sedumoside G or isomer | 17950214 | 0 |
| 205 | 26.95 | [M-H]^-^ | 431.0986 | 431.0984 | 0.468 | C21H20O10 | Apigenin-7-O-β-D-glucopyranoside or isomer | 18380526 | 0 |
| 206 | 27.28 | [M-H]^-^ | 269.0458 | 269.0455 | 0.854 | C15H10O5 | 7,3',4'-Trimethoxy luteolin/7,3',4'-Trihydroxyflavone/Apigenin or isomer | 18558425 | 0 |
| 207 | 27.49 | [M-H]^-^ | 343.0826 | 343.0823 | 0.831 | C18H16O7 | 3,7,4'-O-Trimethylquercetin/3,3',4'-Trimethylquercetin or isomer | 16272620 | 0 |
| 208 | 27.92 | [M-H]^-^ | 281.1396 | 281.1394 | 0.391 | C15H22O5 | 8β,9β-Dihydroxy-1β,10α-epoxy-11β,13-dihydrocostunolide or isomer | 72307640 | 398452 |
| 209 | 27.99 | [M-H]^-^ | 315.0515 | 315.0510 | 1.645 | C16H12O7 | 3-Methylquercetin/Isorhamnetin/Isorhamnetin/Rhamnetin or isomer | 8046979 | 0 |
| 210 | 28.11 | [M-H]^-^ | 345.0618 | 345.0616 | 0.601 | C17H14O8 | Limocitrin or isomer | 15648566 | 0 |
| 211 | 28.19 | [M-H]^-^ | 329.0670 | 329.0667 | 0.850 | C17H14O7 | 3,7-Dimethylquercetin/3,4'-O-Dimethylquercetin/3',4'-Dimethoxy quercetin/3,3'-Dimethoxy quercetin/Tricin | 31871714 | 0 |
| 212 | 28.24 | [M-H]^-^ | 255.0662 | 255.0663 | -0.291 | C15H12O4 | Liquiritigenin/Isoliquiritigenin or isomer | 9080922 | 0 |
| 213 | 28.37 | [M+H]^+^ | 211.0683 | 211.0866 | -1.282 | C13H10N2O | l-Acetyl-β-carboline | 19588873 | 0 |
| 214 | 28.60 | [M+H]^+^ | 301.0701 | 301.0707 | -1.879 | C16H12O6 | Kaempferide or isomer | 33488611 | 276779 |
| 215 | 28.68 | [M+H]^+^ | 191.1064 | 191.1067 | -1.503 | C12H14O2 | Ethyl 4-methylcinnamate or isomer | 31395490 | 1048787 |
| 216 | 28.85 | [M+H]^+^ | 337.2366 | 337.2373 | -2.084 | C20H32O4 | Ent-15,16,18-trihydroxy-2-oxo-pimar-8(14)-ene/Ent-2-oxo-15,16,19-trihydroxypimar-8(14)-ene/Ent-15-oxo-2β,16,19-trihydroxypimar-8(14)-ene/Ent-12α,16-epoxy-2β,15α,19-trihydroxypimar-8(14)-ene/Ent-12α,16-epoxy-2β,15α,19-trihydroxypimar-8-ene/Ent-12α,16-epoxy-2β,15α,19-trihydroxypimar-8-ene/Ent-14β,16-epoxy-8-pimar-ene-2α,15α,19-triol/Ent-16αH,17-hydroxy-kauran-19-oic acid/Siegesbeckic acid/Ent-16β,17-dihydroxykauran-19-oic acid/Ent-17α,18-dihydroxy-kauran-19-oic acid | 644803105 | 0 |
| 217 | 28.85 | [M+H]^+^ | 319.2262 | 319.2268 | -1.801 | C20H30O3 | Grandifloric acid/18-Hydroxy- kauran-16-en-19-oic acid or isomer | 155923034 | 0 |
| 218 | 28.87 | [M+H]^+^ | 499.2894 | 499.2902 | -1.586 | C26H42O9 | Pubeside C/Pubeside D/Ent-2-oxo-3β,15,16-trihydroxypimar-8(14)-en-3-O-β-glucopyranoside | 117221096 | 0 |
| 219 | 29.28 | [M-H]^-^ | 269.0457 | 269.0455 | 0.400 | C15H10O5 | 7,3',4'-Trimethoxy luteolin/7,3',4'-Trihydroxyflavone/Apigenin or isomer | 89903334 | 0 |
| 220 | 29.32 | [M-H]^-^ | 281.1396 | 281.1394 | 0.499 | C15H22O5 | 8β,9β-Dihydroxy-1β,10α-epoxy-11β,13-dihydrocostunolide or isomer | 10901850 | 0 |
| 221 | 29.56 | [M+H]^+^ | 319.2263 | 319.2268 | -1.515 | C20H30O3 | Grandifloric acid/18-Hydroxy- kauran-16-en-19-oic acid or isomer | 71772731 | 2428420 |
| 222 | 30.06 | [M+H]^+^ | 185.1534 | 185.1536 | -0.968 | C11H20O2 | 5-Heptyldihydro-3-furanone or isomer | 4782992 | 0 |
| 223 | 30.08 | [M+H]^+^ | 245.1743 | 245.1747 | -1.835 | C13H24O4 | Sarmentoic acid or isomer | 36682281 | 609014 |
| 224 | 30.10 | [M+H]^+^ | 485.3102 | 485.3109 | -1.534 | C26H44O8 | Darutoside/Pubeside A/Pubeside B/Siegesbeckioside | 14280059 | 0 |
| 225 | 30.31 | [M+H]^+^ | 319.2263 | 319.2268 | -1.610 | C20H30O3 | Grandifloric acid/18-Hydroxy- kauran-16-en-19-oic acid or isomer | 103229212 | 0 |
| 226 | 30.33 | [M+H]^+^ | 337.2367 | 337.2373 | -1.903 | C20H32O4 | Ent-15,16,18-trihydroxy-2-oxo-pimar-8(14)-ene/Ent-2-oxo-15,16,19-trihydroxypimar-8(14)-ene/Ent-15-oxo-2β,16,19-trihydroxypimar-8(14)-ene/Ent-12α,16-epoxy-2β,15α,19-trihydroxypimar-8(14)-ene/Ent-12α,16-epoxy-2β,15α,19-trihydroxypimar-8-ene/Ent-12α,16-epoxy-2β,15α,19-trihydroxypimar-8-ene/Ent-14β,16-epoxy-8-pimar-ene-2α,15α,19-triol/Ent-16αH,17-hydroxy-kauran-19-oic acid/Siegesbeckic acid/Ent-16β,17-dihydroxykauran-19-oic acid/Ent-17α,18-dihydroxy-kauran-19-oic acid | 452047211 | 1934941 |
| 227 | 30.53 | [M+H]^+^ | 379.2471 | 379.2479 | -2.189 | C22H34O5 | 2-Keto-16-acetyloxykirenol/Ent-18-acetoxy-17-hydroxy-16βH-kauran-19-oic acid or isomer | 20532392 | 0 |
| 228 | 30.70 | [M+H]^+^ | 339.2522 | 339.2530 | -2.235 | C20H34O4 | Kirenol/Ent-2α,15,16,19-tetrahydroxypimar-8(14)-ene/7β-Hydroxydarutigenol/9β-Hydroxydarutigenol | 44912362 | 0 |
| 229 | 30.70 | [M+H]^+^ | 321.2418 | 321.2424 | -2.058 | C20H32O3 | Ent-15,16-dihydroxy-2-oxo-pimar-8(14)-ene/Ent-14β,16-epoxy-8-pimar-ene-3β,15α-diol/Ent-16βH,17-hydroxy-kauran-19-oic acid | 2284297926 | 7835331 |
| 230 | 30.92 | [M-H]^-^ | 335.2230 | 335.2228 | 0.715 | C20H32O4 | Ent-15,16,18-trihydroxy-2-oxo-pimar-8(14)-ene/Ent-2-oxo-15,16,19-trihydroxypimar-8(14)-ene/Ent-15-oxo-2β,16,19-trihydroxypimar-8(14)-ene/Ent-12α,16-epoxy-2β,15α,19-trihydroxypimar-8(14)-ene/Ent-12α,16-epoxy-2β,15α,19-trihydroxypimar-8-ene/Ent-12α,16-epoxy-2β,15α,19-trihydroxypimar-8-ene/Ent-14β,16-epoxy-8-pimar-ene-2α,15α,19-triol/Ent-16αH,17-hydroxy-kauran-19-oic acid/Siegesbeckic acid/Ent-16β,17-dihydroxykauran-19-oic acid/Ent-17α,18-dihydroxy-kauran-19-oic acid | 337259497 | 3019869 |
| 231 | 30.97 | [M+H]^+^ | 185.1534 | 185.1536 | -0.968 | C11H20O2 | 5-Heptyldihydro-3-furanone or isomer | 4586406 | 0 |
| 232 | 31.25 | [M-H]^-^ | 389.1620 | 389.1606 | 3.626 | C21H26O7 | Siegenolides A | 11466038 | 0 |
| 233 | 31.54 | [M-H]^-^ | 379.2493 | 379.2490 | 0.793 | C22H36O5 | Orientalin A/Orientalin B/19-Acetoxy-15-hydroxy-12-oxo-13,14E-dehydro-10,11,14,15-tetrahydrogeranylnerol | 36835301 | 0 |
| 234 | 31.70 | [M+H]^+^ | 485.3095 | 485.3109 | -2.855 | C26H44O8 | Darutoside/Pubeside A/Pubeside B/Siegesbeckioside | 7435484 | 0 |
| 235 | 31.70 | [M+H]^+^ | 323.2574 | 323.2581 | -1.934 | C20H34O3 | Ent-2β,15,16-trihydroxy-pimar-8(14)-ene/Darutigenol/Siegesbeckiol/Ent-kauran-16β,17,18-triol | 78746241 | 0 |
| 236 | 31.94 | [M-H]^-^ | 351.2180 | 351.2177 | 0.738 | C20H32O5 | Ent-2β,15,16-trihydroxypimar-8(14)-en-19-oic acid/Ent-16β,17,18-trihydroxy-kauran-19-oic acid | 126910510 | 2274486 |
| 237 | 32.03 | [M+H]^+^ | 285.0752 | 285.0758 | -1.946 | C16H12O5 | Glycitein or isomer | 73080262 | 420053 |
| 238 | 32.04 | [M-H]^-^ | 379.2492 | 379.2490 | 0.471 | C22H36O5 | Orientalin A/Orientalin B/19-Acetoxy-15-hydroxy-12-oxo-13,14E-dehydro-10,11,14,15-tetrahydrogeranylnerol | 14655762 | 0 |
| 239 | 32.17 | [M-H]^-^ | 393.2286 | 393.2283 | 0.800 | C22H34O6 | Ent-18-acetoxy-16α,17-dihydroxykauran-19-oic acid | 181772471 | 0 |
| 240 | 32.24 | [M+H]^+^ | 379.2471 | 379.2479 | -2.029 | C22H34O5 | 2-Keto-16-acetyloxykirenol/Ent-18-acetoxy-17-hydroxy-16βH-kauran-19-oic acid or isomer | 16257801 | 0 |
| 241 | 32.42 | [M-H]^-^ | 329.0660 | 329.0667 | 0.479 | C17H14O7 | 3,7-Dimethylquercetin/3,4'-O-Dimethylquercetin/3',4'-Dimethoxy quercetin/3,3'-Dimethoxy quercetin/Tricin | 104986601 | 0 |
| 242 | 32.69 | [M-H]^-^ | 525.3071 | 525.3069 | 0.308 | C28H46O9 | Hythiemoside B/16-O-Acetyldarutoside or isomer | 33371599 | 0 |
| 243 | 32.95 | [M+H]^+^ | 381.2628 | 381.2636 | -2.084 | C22H36O5 | Orientalin A/Orientalin B/19-Acetoxy-15-hydroxy-12-oxo-13,14E-dehydro-10,11,14,15-tetrahydrogeranylnerol | 46377025 | 0 |
| 244 | 33.04 | [M-H]^-^ | 525.3073 | 525.3069 | 0.656 | C28H46O9 | Hythiemoside B/16-O-Acetyldarutoside or isomer | 21977351 | 0 |
| 245 | 33.34 | [M+H]^+^ | 285.0752 | 285.0758 | -2.053 | C16H12O5 | Glycitein or isomer | 16593510 | 243281 |
| 246 | 33.43 | [M+H]^+^ | 245.1745 | 245.1747 | -0.839 | C13H24O4 | Sarmentoic acid or isomer | 5773533 | 0 |
| 247 | 33.53 | [M+H]^+^ | 403.1382 | 403.1387 | -1.466 | C21H22O8 | 5,6,7,3',4',5'-Pentamethoxyflavone | 11980723 | 0 |
| 248 | 34.65 | [M+H]^+^ | 365.2679 | 365.2686 | -1.894 | C22H36O4 | 16-O-Acetyldarutigenol or isomer | 18388710 | 0 |
| 249 | 34.67 | [M+H]^+^ | 527.3198 | 527.3215 | -3.101 | C28H46O9 | Hythiemoside B/16-O-Acetyldarutoside or isomer | 10130884 | 0 |
| 250 | 34.98 | [M+H]^+^ | 345.0963 | 345.0969 | -1.640 | C18H16O7 | 3,7,4'-O-Trimethylquercetin/3,3',4'-Trimethylquercetin or isomer | 41789771 | 0 |
| 251 | 35.00 | [M-H]^-^ | 335.2230 | 335.2228 | 0.533 | C20H32O4 | Ent-15,16,18-trihydroxy-2-oxo-pimar-8(14)-ene/Ent-2-oxo-15,16,19-trihydroxypimar-8(14)-ene/Ent-15-oxo-2β,16,19-trihydroxypimar-8(14)-ene/Ent-12α,16-epoxy-2β,15α,19-trihydroxypimar-8(14)-ene/Ent-12α,16-epoxy-2β,15α,19-trihydroxypimar-8-ene/Ent-12α,16-epoxy-2β,15α,19-trihydroxypimar-8-ene/Ent-14β,16-epoxy-8-pimar-ene-2α,15α,19-triol/Ent-16αH,17-hydroxy-kauran-19-oic acid/Siegesbeckic acid/Ent-16β,17-dihydroxykauran-19-oic acid/Ent-17α,18-dihydroxy-kauran-19-oic acid | 206664504 | 0 |
| 252 | 35.06 | [M-H]^-^ | 281.1394 | 281.1394 | -0.152 | C15H22O5 | 8β,9β-Dihydroxy-1β,10α-epoxy-11β,13-dihydrocostunolide or isomer | 10944914 | 0 |
| 253 | 35.58 | [M-H]^-^ | 363.2544 | 363.2541 | 0.858 | C22H36O4 | 16-O-Acetyldarutigenol or isomer | 17002536 | 0 |
| 254 | 36.13 | [M+H]^+^ | 485.3099 | 485.3109 | -2.037 | C26H44O8 | Darutoside/Pubeside A/Pubeside B/Siegesbeckioside | 12011379 | 0 |
| 255 | 36.13 | [M+H]^+^ | 323.2573 | 323.2581 | -2.312 | C20H34O3 | Ent-2β,15,16-trihydroxy-pimar-8(14)-ene/Darutigenol/Siegesbeckiol/Ent-kauran-16β,17,18-triol | 209317160 | 935774 |
| 256 | 36.35 | [M-H]^-^ | 367.1187 | 367.1187 | -0.245 | C21H20O6 | Curcumin | 4127947 | 0 |
| 257 | 36.37 | [M-H]^-^ | 525.3070 | 525.3069 | 0.192 | C28H46O9 | Hythiemoside B/16-O-Acetyldarutoside or isomer | 10971831 | 0 |
| 258 | 36.69 | [M+H]^+^ | 323.2571 | 323.2581 | -2.972 | C20H34O3 | Ent-2β,15,16-trihydroxy-pimar-8(14)-ene/Darutigenol/Siegesbeckiol/Ent-kauran-16β,17,18-triol | 38323648 | 0 |
| 259 | 37.07 | [M-H]^-^ | 377.2334 | 377.2333 | 0.216 | C22H34O5 | 2-Keto-16-acetyloxykirenol/Ent-18-acetoxy-17-hydroxy-16βH-kauran-19-oic acid or isomer | 104629541 | 0 |
| 260 | 37.37 | [M+H]^+^ | 365.2679 | 365.2686 | -1.977 | C22H36O4 | 16-O-Acetyldarutigenol or isomer | 12341284 | 0 |
| 261 | 38.37 | [M+H]^+^ | 527.3190 | 527.3215 | -4.606 | C28H46O9 | Hythiemoside B/16-O-Acetyldarutoside or isomer | 3250686 | 0 |
| 262 | 38.39 | [M+H]^+^ | 365.2677 | 365.2686 | -2.562 | C22H36O4 | 16-O-Acetyldarutigenol or isomer | 7159824 | 0 |
| 263 | 38.53 | [M+H]^+^ | 235.1689 | 235.1693 | -1.648 | C15H22O2 | Germacranolide/Curcumenol or isomer | 68463759 | 0 |
| 264 | 38.84 | [M-H]^-^ | 233.1543 | 233.1547 | -1.612 | C15H22O2 | Germacranolide/Curcumenol or isomer | 8381669 | 0 |
| 265 | 38.89 | [M+H]^+^ | 365.2679 | 365.2686 | -1.977 | C22H36O4 | 16-O-Acetyldarutigenol or isomer | 7071080 | 0 |
| 266 | 39.60 | [M-H]^-^ | 269.0456 | 269.0455 | 0.173 | C15H10O5 | 7,3',4'-Trimethoxy luteolin/7,3',4'-Trihydroxyflavone/Apigenin or isomer | 22690990 | 0 |
| 267 | 39.99 | [M-H]^-^ | 319.2279 | 319.2279 | 0.212 | C20H32O3 | Ent-15,16-dihydroxy-2-oxo-pimar-8(14)-ene/Ent-14β,16-epoxy-8-pimar-ene-3β,15α-diol/Ent-16βH,17-hydroxy-kauran-19-oic acid | 26015864 | 0 |
| 268 | 40.35 | [M-H]^-^ | 463.2705 | 463.2701 | 0.523 | C26H40O7 | Ent-18-acetoxy-16α-hydroxy-17-isobutyryloxykauran-19-oic acid | 15646724 | 0 |
| 269 | 41.07 | [M-H]^-^ | 317.2122 | 317.2122 | -0.093 | C20H30O3 | Grandifloric acid/18-Hydroxy- kauran-16-en-19-oic acid or isomer | 72595011 | 0 |
| 270 | 41.23 | [M+H]^+^ | 245.1743 | 245.1747 | -1.710 | C13H24O4 | Sarmentoic acid or isomer | 3885655 | 0 |
| 271 | 41.28 | [M-H]^-^ | 319.2278 | 319.2279 | -0.266 | C20H32O3 | Ent-15,16-dihydroxy-2-oxo-pimar-8(14)-ene/Ent-14β,16-epoxy-8-pimar-ene-3β,15α-diol/Ent-16βH,17-hydroxy-kauran-19-oic acid | 89153091 | 0 |
| 272 | 41.32 | [M-H]^-^ | 233.1543 | 233.1547 | -1.924 | C15H22O2 | Germacranolide/Curcumenol or isomer | 5311591 | 0 |
| 273 | 41.43 | [M-H]^-^ | 321.2433 | 321.2435 | -0.722 | C20H34O3 | Ent-2β,15,16-trihydroxy-pimar-8(14)-ene/Darutigenol/Siegesbeckiol/Ent-kauran-16β,17,18-triol | 8673886 | 0 |
| 274 | 41.57 | [M-H]^-^ | 249.1496 | 249.1496 | -0.818 | C15H22O3 | 2-Desoxy-4-epi-pulchellin | 237892287 | 0 |
| 275 | 41.60 | [M-H]^-^ | 447.2754 | 447.2752 | 0.331 | C26H40O6 | Siegesesteric acid/Ent-16βH,17-acetoxy-18-isobutyryloxykauran-19-oic acid/Ent-18-acetoxy-17-isobutyryloxy-16βH-kauran-19-oic acid | 15534544 | 0 |
| 276 | 41.78 | [M-H]^-^ | 447.2755 | 447.2752 | 0.740 | C26H40O6 | Siegesesteric acid/Ent-16βH,17-acetoxy-18-isobutyryloxykauran-19-oic acid/Ent-18-acetoxy-17-isobutyryloxy-16βH-kauran-19-oic acid | 6714411 | 0 |
| 277 | 41.93 | [M-H]^-^ | 213.1853 | 213.1860 | -3.072 | C13H26O2 | ( 3S,5R,6S,9R)-Megastigmane-3,9-diol or isomer | 17642445 | 0 |
| 278 | 42.07 | [M-H]^-^ | 389.2701 | 389.2697 | 0.893 | C24H38O4 | Ent-16βH,17-isobutyryloxy-kauran-19-oic acid or isomer | 6718557 | 0 |
| 279 | 42.32 | [M-H]^-^ | 213.1855 | 213.1860 | -2.213 | C13H26O2 | ( 3S,5R,6S,9R)-Megastigmane-3,9-diol or isomer | 8879275 | 0 |
| 280 | 42.45 | [M-H]^-^ | 279.2330 | 279.2330 | 0.282 | C18H32O2 | 9,12-Octadecadiynoic acid | 128867798 | 0 |
| 281 | 42.64 | [M-H]^-^ | 213.1853 | 213.1860 | -3.215 | C13H26O2 | ( 3S,5R,6S,9R)-Megastigmane-3,9-diol or isomer | 14067679 | 0 |
| 282 | 42.70 | [M-H]^-^ | 305.2490 | 305.2486 | 1.176 | C20H34O2 | Ent-kauran-19β,17-diol | 2176561 | 0 |
| 283 | 42.90 | [M-H]^-^ | 213.1853 | 213.1860 | -3.286 | C13H26O2 | ( 3S,5R,6S,9R)-Megastigmane-3,9-diol or isomer | 6470019 | 0 |
| 284 | 43.04 | [M+H]^+^ | 391.2835 | 391.2843 | -1.988 | C24H38O4 | Ent-16βH,17-isobutyryloxy-kauran-19-oic acid or isomer | 27105967 | 0 |
| 285 | 43.18 | [M-H]^-^ | 213.1853 | 213.1860 | -3.501 | C13H26O2 | ( 3S,5R,6S,9R)-Megastigmane-3,9-diol or isomer | 1979638 | 0 |
